# Structural basis for phosphatidylinositol-phosphate biosynthesis

**DOI:** 10.1038/ncomms9505

**Published:** 2015-10-16

**Authors:** Oliver B. Clarke, David Tomasek, Carla D. Jorge, Meagan Belcher Dufrisne, Minah Kim, Surajit Banerjee, Kanagalaghatta R. Rajashankar, Lawrence Shapiro, Wayne A. Hendrickson, Helena Santos, Filippo Mancia

**Affiliations:** 1 Department of Biochemistry and Molecular Biophysics, Columbia University, New York, NY 10032, USA; 2 Department of Physiology and Cellular Biophysics, Columbia University, New York, NY 10032, USA; 3 Biology Division, Instituto de Tecnologia Química e Biológica, Universidade Nova de Lisboa, Avenida da República-EAN, 2780-157 Oeiras, Portugal; 4 NE-CAT and Department of Chemistry and Chemical Biology, Cornell University, Argonne National Laboratory, Argonne, IL 60439, USA

## Abstract

Phosphatidylinositol is critical for intracellular signalling and anchoring of carbohydrates and proteins to outer cellular membranes. The defining step in phosphatidylinositol biosynthesis is catalysed by CDP-alcohol phosphotransferases, transmembrane enzymes that use CDP-diacylglycerol as donor substrate for this reaction, and either inositol in eukaryotes or inositol phosphate in prokaryotes as the acceptor alcohol. Here we report the structures of a related enzyme, the phosphatidylinositol-phosphate synthase from *Renibacterium salmoninarum*, with and without bound CDP-diacylglycerol to 3.6 and 2.5 Å resolution, respectively. These structures reveal the location of the acceptor site, and the molecular determinants of substrate specificity and catalysis. Functional characterization of the 40%-identical ortholog from *Mycobacterium tuberculosis*, a potential target for the development of novel anti-tuberculosis drugs, supports the proposed mechanism of substrate binding and catalysis. This work therefore provides a structural and functional framework to understand the mechanism of phosphatidylinositol-phosphate biosynthesis.

## Abstract (web summary)

CDP-alcohol phosphotransferases (CDP-APs) are critical for the biosynthesis of glycerophospholipids. Here, Clarke *et al.* present the first structure of an enzymatically active CDP-AP in the presence of a bound lipid substrate and propose a mechanism for substrate binding and catalysis.

## Graphical abstract

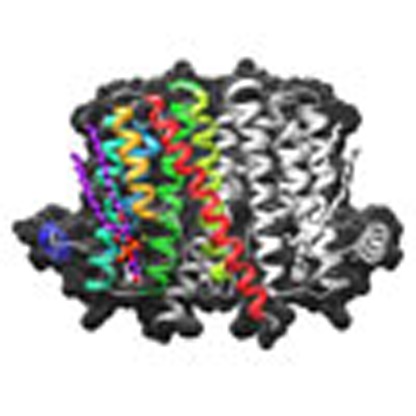

In eukaryotes, phosphatidylinositol (PI)-based lipids (phosphoinositides) play important roles in numerous aspects of intracellular signalling and in the anchoring of glycosylphosphatidylinositol-linked proteins to the membrane. In prokaryotes, PI is produced by mycobacteria, as well as some other bacterial genera, where it is required for the biosynthesis of key components of the cell wall. For example, the cell walls of mycobacteria carry complex lipoglycans such as lipomannan and lipoarabinomannan, which are tethered to the membrane via a common PI anchor that constitutes their first building block^1^. In *Mycobacterium tuberculosis*, these lipids function as important virulence factors and modulators of the host immune response^1^,^2^.

Eukaryotic PI synthases process *myo-*inositol and CDP-diacylglycerol (CDP-DAG) to generate PI directly in a single step^3^. In prokaryotes, PI biosynthesis occurs in two steps. In the initial step, phosphatidylinositol-phosphate (PIP) synthases (Fig. 1a) generate PIP using L-*myo*-inositol-1-phosphate (inositol phosphate) as the acceptor alcohol and CDP-DAG as the donor substrate; subsequently, the terminal phosphate is removed by an as-yet-unidentified phosphatase to yield PI^4^. A homologous enzyme (archaetidyl-myo-inositol phosphate synthase) has been characterized in archaea, which like the PIP synthases uses inositol phosphate as the acceptor, but requires CDP-archaeol, an isoprene-based ether-linked lipid, as the donor instead of CDP-DAG^5^. Each of these enzymes is a member of the class I CDP-alcohol phosphotransferases (CDP-APs); class II CDP-APs are peripheral membrane proteins of an unrelated fold, and are not involved in PI biosynthesis (class II enzymes will not be discussed here, and we will use the term CDP-APs as referred to class I family members only). All (class I) CDP-APs are integral membrane enzymes that catalyse the transfer of a substituted phosphate group from a CDP-linked donor, CDP-DAG for PI and PIP biosynthesis, to an acceptor alcohol to generate a phosphodiester-linked product^3^,^6^,^7^.

Two recent structure reports on CDP-APs from *Archaeoglobus fulgidus* (*Af*), a protein termed *Af*2299 and a di-*myo*-inositol-phosphate phosphate synthase (*Af*DIPP synthase) have offered a first glimpse of the transmembrane (TM) architecture and catalytic machinery of this enzyme family^8^,^9^. However, neither *Af*2299 nor *Af*DIPP synthase process lipids, leaving unanswered the key question of how membrane-embedded substrates are recruited and processed by CDP-APs, to generate glycerophospholipids such as PI and PIP. To shed light on this question at a molecular level, we decided to focus on the PIP synthases for three reasons. First, they are the closest evolutionary relatives to *Af*2299 and *Af*DIPP synthase^10^, an advantage for the crystal engineering approach we adopted here and which is described below. Second, PIP synthases bind CDP-DAG as donor substrate, a feature in common with eukaryotic PI and cardiolipin synthases, as well as all prokaryotic CDP-APs involved in glycerophospholipid biosynthesis. Third, they have possible medical relevance, as genetic ablation of mycobacterial PIP synthase is lethal^11^. This, combined with the unique pathway used for PI synthesis in prokaryotes, may position PIP synthase as a potentially attractive future drug target^12^,^13^.

We have determined the crystal structures of PIP synthase from *Renibacterium salmoninarum,* in the apo form and with bound CDP-DAG to 2.5 and 3.6 Å resolution, respectively. These structures show how CDP-DAG binds to the enzyme, and reveal the molecular determinants of substrate specificity and catalysis. Functional assays performed on PIP synthase from *M. tuberculosis*, which is 40%-identical to the ortholog from *R. salmoninarum*, and a potential target for the development of novel anti-tuberculosis drugs, supports the proposed mechanism of substrate binding and catalysis. This work provides both a structural and a functional framework to investigate and understand PIP biosynthesis.

## Results

### Crystal engineering and structure determination

Initial attempts to express and crystallize several mycobacterial PIP synthases and close bacterial homologues were unsuccessful. We rationalized that this might be due, at least in part, to the lack of a crystal contact forming soluble domain. Unlike most other CDP-APs, both *Af*2299 and *Af*DIPP synthase have an N-terminal cytosolic cytidylyltransferase-like domain^14^,^15^, which provided the essential contacts in the crystal lattice between layers of molecules^8^. We reasoned that the *Af*2299 extramembrane domain might be able to improve the performance in crystallization experiments of other CDP-APs. To test this hypothesis, we generated chimeric constructs by fusing the *Af*2299 cytosolic domain to the N-terminus of 14 different PIP synthases. We also introduced mutations at six positions in all 14 PIP-synthase sequences to mimic the interface between soluble and TM domains observed in the structure of *Af*2299 (Supplementary Fig. 1). Attachment of this domain led to a significant increase in expression levels for all the constructs tested, and proteins from several species of mycobacteria and bacteria yielded crystals by the lipidic cubic phase technique^16^. We determined the structure of a chimeric construct of PIP synthase from *R. salmoninarum* (*Rs*PIPS)—the causative agent for bacterial kidney disease in salmonids, a major threat to these species worldwide^17^—in which the first six residues of the *Rs*PIPS sequence were omitted (*Rs*PIPS-Δ6N; Supplementary Fig. 1). Crystals of *Rs*PIPS-Δ6N diffracted X-rays to 2.5 Å resolution, and the structure was solved by molecular replacement using the cytidylyltransferase-like domain of *Af*2299 as a search model (Table 1). Subsequently, we determined the structure of a construct containing the complete *Rs*PIPS sequence (absent the initiating methionine; *Rs*PIPS-FL) in complex with CDP-DAG at 3.6 Å resolution, again by molecular replacement, in this instance using *Rs*PIPS-Δ6N as search model (Table 1 and Supplementary Fig. 1).

In both structures, the relative disposition of the transmembrane and soluble domains is very similar to that observed in the structure of *Af*2299. Of the six mutations introduced at the *Rs*PIPS–*Af*2299 interface, two appear particularly important in limiting flexibility between the two domains. L75 and F77, which are both located in the loop between TM2 and TM3, are buried in hydrophobic pockets on the surface of the cytidylyltransferase-like domain, replicating the interactions observed in the crystal structure of *Af*2299 (Supplementary Fig. 1).

### Transmembrane architecture and active site in *Rs*PIPS-Δ6N

**Figure 1:**
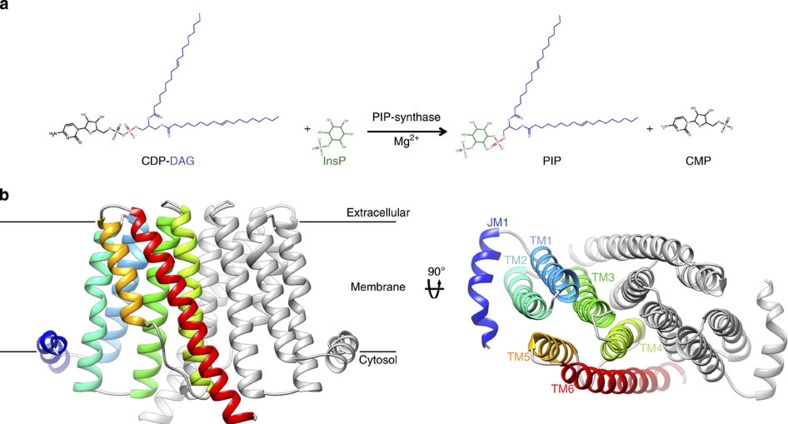
Transmembrane architecture of a PIP synthase from *R. salmoninarum*. (**a**) PIP synthases catalyse the transfer of a diacylglycerol-substituted phosphate group (purple/red) from the CDP-DAG donor to the inositol phosphate acceptor (green), generating PIP and CMP. (**b**) Structure of the *Rs*PIPS-Δ6N homodimer in ribbon representation viewed from two orthogonal orientations (in the plane of the membrane on the left; towards the cytosol down the dimer axis on the right). One protomer is coloured grey, and the helices of the other are depicted in spectral colouring, from blue (JM1) to red (TM6). The *Af*2299 extramembrane domain used to facilitate crystallization is not shown here.

**Figure 2:**
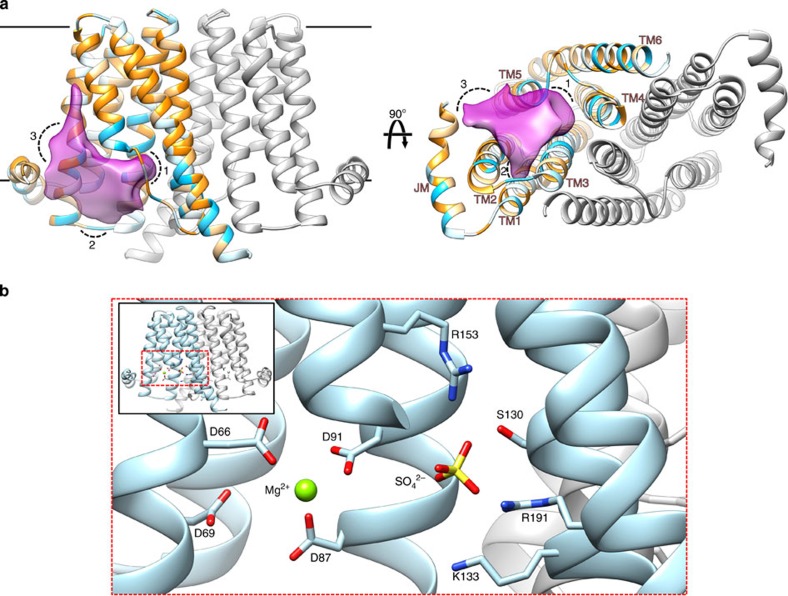
A large interfacial cavity contains the active site of *Rs*PIPS. (**a**) The structure of *Rs*PIPS-Δ6N is shown in ribbon representation, with one protomer coloured grey and the other coloured by the Kyte–Doolitle hydrophobicity scale^34^, from −4.5 (most polar, light blue) to 4.5 (most hydrophobic, orange). Two orthogonal representations are shown, on the left is a view in the plane of the membrane and on the right is a view from the cytosol along the dimer axis. A transparent purple surface (calculated using the 3V server^35^) delineates the borders of the interfacial cavity, which contains three subregions as follows: 1, the inositol phosphate acceptor-binding pocket; 2, the nucleotide-binding pocket between TM2 and TM3; and 3, a hydrophobic groove between TM2 and JM1. (**b**) Detail of the active site viewed in the plane of the membrane, with side chains that contact the bound Mg^2+^ and 
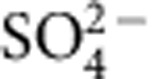
 ions labelled and depicted in stick representation.

*Rs*PIPS adopts a homodimeric architecture similar to those previously observed in *Af*2299 and *Af*DIPP synthase (Fig. 1b and Supplementary Fig. 2), with each protomer possessing six TM helices surrounding a large polar cavity. Sequence alignment with eukaryotic CDP-APs that process a lipidic acceptor substrate, such as choline/ethanolamine phosphotransferase (CEPT1; Supplementary Fig. 3) suggests that these eukaryotic enzymes may possess an additional three to four TM helices at the C terminus, perhaps serving as an additional TM module to accommodate the bulkier, hydrophobic acceptor. In *Rs*PIPS, the central polar cavity is located at the cytosolic boundary of the membrane, and contains three distinct regions, which together form the active site (Fig. 2a). The nucleotide-binding site is delineated by TMs 1, 2 and 3, and is characterized by a signature sequence featuring eight absolutely conserved residues (D_1_xxD_2_G_1_xxAR…G_2_xxxD_3_xxxD_4_) (ref. 18), five of which are located on TM2 and three on TM3. As observed previously^8^, the first three of the conserved aspartate side chains coordinate a metal, and D_4_ likely acts as the catalytic base. The four other signature amino acids either provide structural flexibility or line the binding site that accommodates the pyrimidine ring of the CDP^8^.

Proximal to the nucleotide-binding site, and within the membrane-spanning region is a pocket wedged between TMs 4, 5 and 6, which probably represents the inositol phosphate acceptor-binding cavity (Fig. 2). Several conserved residues line this cavity, including two arginine residues (R153 and R191) that in the structure of *Rs*PIPS-Δ6N coordinate a 
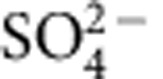
 ion present at high concentration in the crystallization solution. R153 and R191 are absolutely conserved amongst all PIP synthases, but not in eukaryotic PI synthases. We hypothesize that these residues coordinate the phosphate of inositol phosphate, a moiety unique to PIP synthases.

Directly above the nucleotide-binding site, we observed a gap between JM1 and TM2, creating a hydrophobic groove that is open to the membrane. In contrast, the structures of *Af*2299 and *Af*DIPP synthase displayed a small, hydrophilic pocket in this region, which in the case of *Af*2299 was shown to accommodate the glycerol moiety of the CDP-glycerol donor. The difference in the nature of the donor substrate, CDP-DAG for *Rs*PIPS and CDP-glycerol for *Af*2299 is most likely responsible for the differences observed in the architecture of the donor-substituent site in these two enzymes.

### Structure of *Rs*PIPS in complex with CDP-DAG

**Figure 3:**
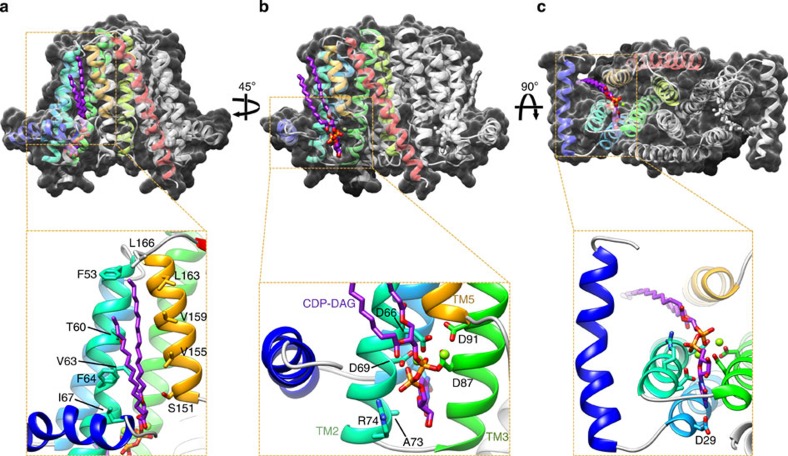
CDP-DAG binding to *Rs*PIPS-FL. The structure of *Rs*PIPS-FL, depicted in ribbon representation and coloured as per Fig. 1, is shown in three different views (**a**–**c**) superimposed on a transparent ‘Stromboli black' spacefill model (upper panels), with matching magnified insets of boxed regions below (lower panels). CDP-DAG is depicted in purple and Mg^2+^ ions in light green. Side chains that contact CDP-DAG or Mg^2+^ are shown in stick representation.

We initially engineered *Rs*PIPS chimeric constructs with alternative junctions to the N-terminal domain (Supplementary Fig. 1), and crystals were obtained of two of these, *R*sPIPS-Δ6N and *Rs*PIPS-FL. The crystals of *Rs*PIPS-Δ6N diffracted well in the apo-state (2.5 Å), but attempts to obtain co-crystal structures with CDP-DAG were unsuccessful. In contrast, although crystals of *Rs*PIPS-FL only diffracted to lower resolution (3.6 Å), a structure of the complex with CDP-DAG was obtained readily. The structure of the complex of CDP-DAG bound to *Rs*PIPS-FL revealed strong density for CDP, with the nucleotide ring wedged between TMs 2 and 3, and the diphosphate moiety coordinated by a bound magnesium ion that also interacts with conserved aspartate residues of the CDP-AP signature sequence (Fig. 2b). In all four protomers in the asymmetric unit, density was also observed for the acyl chains of the CDP-DAG, which lie against the TM region in a groove formed by JM1, TM2 and TM5 (Fig. 3, Supplementary Fig. 4 and Supplementary Movie 1). This groove is entirely absent in the two previous structures of CDP-APs, which have a small, hydrophilic pocket in this location, consistent with their preference for soluble donor moieties such as CDP-glycerol and CDP-inositol (Supplementary Fig. 1i).

### Functional validation of crystallization constructs

Functional characterization of *Rs*PIPS-FL, performed by measuring incorporation of L-*myo*-[^14^C]inositol-1-phosphate into membranes derived from *Rs*PIPS-FL-expressing *Escherichia coli* cells, revealed that although this enzyme from *R. salmoninarum* exhibits significant Mg^2+^-dependent PIP-synthase activity (Supplementary Fig. 1j), we judged the activity level to be too low to provide the basis for a reliable assay system. By contrast, equivalent constructs of the close PIP-synthase homolog from *M. tuberculosis* (*Mt*PIPS; 40% identity to *Rs*PIPS) showed robust specific activity (Supplementary Fig. 1j). We therefore used *Mt*PIPS, which has high homology to *Rs*PIPS in its active site region (Supplementary Fig. 5), as an assay system for structure-based functional characterization of PIP synthases.

Functional characterization of the chimeric *Mt*PIPS proteins revealed that the activity of *Mt*PIPS-Δ6N was substantially lower than that of *Mt*PIPS-FL (Supplementary Fig. 1j). We hypothesize that this diminished activity of *Mt*PIPS-Δ6N is due to compromised binding of CDP-DAG, as JM1 is truncated and distorted in *Rs*PIPS-Δ6N, potentially interfering with CDP-DAG binding (Supplementary Fig. 1i). This could provide an explanation as to why we were unable to obtain the structure of *Rs*PIPS-Δ6N in complex with its cognate lipid substrate. The length of the linker appears to be the primary cause of the reduced activity of the Δ6N construct, not the addition of the *Af*2299 domain, nor the interface mutations. Indeed, the activity of *Mt*PIPS-FL is comparable to the activity of *Mt*PIPS constructs lacking the extramembrane domain and interface mutations (Supplementary Fig. 1j). All proteins tested expressed to comparable levels. Kinetic characterization of the construct lacking the extramembrane domain and interface mutations showed that the *K*_M_ for inositol phosphate is somewhat lower for the engineered construct (122 versus 243 μM), while the *K*_M_ for CDP-DAG is somewhat higher (238 versus 60 μM; Supplementary Fig. 6a–d). Importantly, the *V*_max_ for the engineered construct is comparable to that for the unmodified protein (22 versus 32 nmol PIP per min per mg protein; Supplementary Fig. 6a–d). These data suggest that our addition of a crystallization chaperone fusion combined with engineering of the interface between the two domains, prerequisites for successful crystallization and structure determination, did not substantially impact the capability of the enzyme to function as compared with the wild-type (WT) protein.

### Functional characterization of PIP synthase

**Figure 4:**
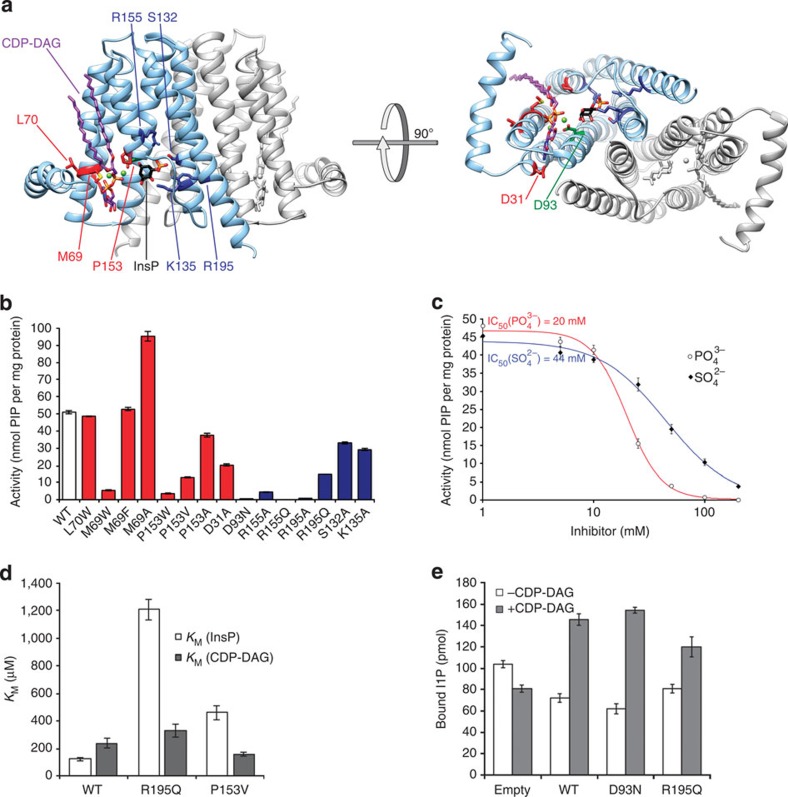
A homology model of *Mt*PIPS, with functional characterization of selected point mutants. (**a**) The *Mt*PIPS homology model is shown in ribbon representation with one protomer in grey and the other in light blue, from two views, on the left viewed in the plane of the membrane and on the right from the cytosol, along the dimer axis. The substrates, CDP-DAG (purple) and inositol phosphate (black), are modelled based on the structures of *Rs*PIPS-FL (in complex with CDP-DAG) and *Rs*PIPS-Δ6N (with bound SO_4_^2−^). The homology model was generated using the Phyre2 server^36^, in one-to-one threading mode using the sequence of *Mt*PIPS (Uniprot accession: P9WPG7) as the target and the structure of *Rs*PIPS-FL (with the *Af*2299 extramembrane domain excised) as the template. Selected residues which are predicted to participate in either inositol phosphate binding (R155, R195, S132, K135), CDP-DAG binding (P153, M69, D31), neither (L70), or catalysis (D93) are shown with side chains in stick representation and coloured as in (**b**), where the activity of point mutants at these positions for the *Mt*PIPS-FL construct is shown compared to wild-type *Mt*PIPS-FL. (**c**) 
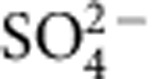
 (closed diamonds) and 
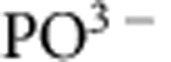
 (open circles) inhibit the activity of *Mt*PIPS-FL with half-inhibitory concentrations of 44 and 22 mM, respectively. (**d**) *K*_M_ of *Mt*PIPS-FL WT, R195Q and P153V for inositol phosphate (InsP; white) and CDP-DAG (grey). InsP, L-*myo*-inositol-1-phosphate (inositol phosphate). (**e**) Quantification of bound L-*myo*-[^14^C]inositol-1-phosphate after incubation of liposomes containing 9 μg *Mt*PIPS-FL (WT, D93N, R195Q or empty liposome control) in the presence and absence of 200 μM CDP-DAG with 40 μM L-*myo*-[^14^C]inositol-1-phosphate. Measurement errors were quantified as s.e.m. (*n*=3).

We selected mutants expected to have compromised substrate binding or catalytic activity based on residue conservation and based on our structures of *Rs*PIPS. These mutations are displayed on a homology model of *Mt*PIPS (Fig. 4a), the enzyme used in our functional assay. All mutants analysed were expressed at levels comparable to WT *Mt*PIPS-FL. Mutation to alanine in *Mt*PIPS of the two arginine residues (R155 and R195) that bind to 
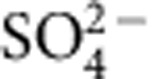
 in the *Rs*PIPS structure (R153 and R191) led to severely compromised activity (Fig. 4b), consistent with disruption of the inositol phosphate-binding site. Both 
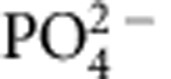
 and 
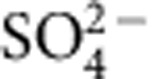
 inhibit activity at concentrations well below those used for crystallization (Fig. 4c), consistent with competition between binding of 
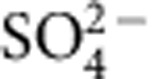
 and the phosphate group of inositol phosphate. More subtle substitutions (R/Q) at the same positions (R155 and R195) also resulted in substantial reduction in enzymatic activity (Fig. 4b). Kinetic characterization of inositol phosphate and CDP-DAG-dependent activity of R195Q (Fig. 4d and Supplementary Fig. 6), which retains ∼40% of WT activity, demonstrated that the mutation had only a mild effect on the *K*_M_ for CDP-DAG (236 μM for WT; 329 μM for R195Q), while severely impairing *K*_M_ for inositol phosphate (122 μM for WT; 1,208 μM for R195Q). Comparison of WT and R195A proteins extracted and purified from isolated membranes with a non-ionic detergent showed that both proteins are membrane inserted and have the same elution profile on size-exclusion chromatography (SEC), strongly suggesting that this point mutation does not compromise folding (Supplementary Fig. 7). Direct single-point measurements of L-*myo*-[^14^C]inositol-1-phosphate binding to liposome-incorporated R195Q, WT and D93N (D_4_) *Mt*PIPS-FL proteins were carried out to differentiate the direct effects of these mutations on inositol phosphate affinity from other mechanisms by which catalysis could be impaired (Fig. 4e). These assays were carried out in the presence and absence of CDP-DAG. Intriguingly, inositol phosphate binding was strictly CDP-DAG dependent (Fig. 4e). Determination of the fraction of [^14^C]PIP in the liposomes after the binding assay was carried out to assess the catalytic activity of the proteins under these conditions. The only sample exhibiting any detectable level of catalytic activity was the WT construct in the presence of CDP-DAG, for which nearly all of the radioactive inositol phosphate above the background level was incorporated in the lipophilic PIP and therefore found in the organic phase (77.8±0.6 pmol per assay; ±indicates standard error of the mean, *n*=3). The amount of inositol phosphate bound in total was comparable to WT for the D93N mutant, consistent with the role of this residue in catalysis, as opposed to substrate recognition. In contrast, R195Q bound a significantly lower amount of substrate, compatible with its putative function in binding of inositol phosphate. Alanine mutagenesis of a conserved serine (S132) that also interacts with the *Rs*PIPS-bound 
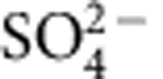
 reduced activity as well, albeit to a lesser extent. Lysine K135 is located such that it may interact with the inositol ring, and the K135A mutation also resulted in a partial loss of activity.

Furthermore, mutation in *Mt*PIPS of P153W, a conserved residue on TM5, which stacks against one of the aliphatic chains of CDP-DAG, resulted in nearly complete loss of enzymatic activity, consistent with a loss of CDP-DAG binding due to obstruction of the lipid-binding groove by the larger tryptophan side chain (Fig. 4a,b). In agreement with this hypothesis, substitution of P153 with alanine had minimal effects on activity, while substitution with valine resulted in a partial defect in activity. Kinetic characterization of P153V (Fig. 4d and Supplementary Fig. 6) showed *V*_max_ to be substantially decreased, consistent with an effect of this mutation on the catalytic efficiency of the enzyme. Unexpectedly, the *K*_M_ for inositol phosphate was increased, whilst the *K*_M_ for CDP-DAG was decreased for this mutant, possibly suggestive of a more complex role of P153, critically located at the CDP-DAG entrance to the active site, and two residues in sequence away from R155, one of the two key inositol phosphate-binding residues. Mutation of another residue, L70W, near the groove but oriented away from it, did not affect the activity (Fig. 4a,b), while substitution of the directly adjacent M69, which contacts CDP-DAG from TM2, by tryptophan resulted in severely impaired activity. Substitution with residues similarly sized or smaller than the native methionine did not compromise activity (Fig. 4a,b).

Not surprisingly, mutation of D31, a conserved residue on TM1, which forms a hydrogen bond with the primary amine of CDP, to alanine, also severely compromised activity, but did not completely abolish it (Fig. 4a,b). This partial effect of the D31A mutation is likely due to the fact that most of the residues in the CDP-AP signature sequence participate in binding of the nucleotide, and that T34, present in all CDP-APs as S or T as part of a conserved P(D/N)xx(T/S) motif, also binds to the primary amine of the pyrimidine ring, and thus may compensate, at least in part, for the absence of the contribution from D31.

Finally and as expected, even a conservative D to N mutation at the site of the putative catalytic base, D93 (the fourth aspartate in the signature sequence) resulted in near complete abrogation of CDP-AP activity (Fig. 4a,b), without compromising substrate binding (Fig. 4e).

## Discussion

The defining step in glycerophospholipid biosynthesis is catalysed by CDP-APs. These constitute a large and diverse family of membrane-embedded enzymes characterized by a signature sequence containing eight absolutely conserved amino acids and 6–10 predicted TM segments. The structure of the CDP-AP *Rs*PIPS reported here exhibits a homodimeric, six-TM architecture similar to those described for *Af*2299 and *Af*DIPP synthase^8^,^9^. This architecture appears to be conserved amongst all the CDP-AP family members that utilize a soluble acceptor substrate such as inositol or inositol phosphate, irrespective of the nature—hydrophobic or hydrophilic—of the CDP-attached donor (Supplementary Fig. 3). These include all characterized prokaryotic CDP-APs, as well as eukaryotic PI synthases. In contrast, CDP-APs that utilize a lipidic acceptor, such as eukaryotic phosphatidylethanolamine (PE) and phosphatidylcholine (PC) synthases, typically have three or four additional TM helices at the C terminus (Supplementary Fig. 3), which are likely required to accommodate the acyl chains of the bulky hydrophobic acceptor substrate.

Fusion of *Rs*PIPS to a crystallization chaperone derived from the extramembrane domain of *Af*2299 was instrumental in obtaining diffracting crystals. The employment of crystallization chaperones is a well-established technique for obtaining crystals of otherwise recalcitrant membrane proteins and has enjoyed particularly extensive use in the field of G-protein-coupled receptor crystallography^19^. We believe that the extramembrane domain of *Af*2299 may prove a valuable addition to the complement of membrane protein crystallization chaperones, although further studies are necessary to demonstrate the general-purpose utility of this fusion partner.

The structure of *Rs*PIPS confirms the locations of the acceptor- and donor-substituent-binding pockets described in the structure of *Af*2299, and identifies a pair of conserved arginine residues (R153 and R191), as involved in the specific recognition of inositol phosphate. Sequence alignment of *Rs*PIPS with human CEPT1 (Supplementary Fig. 3), a CDP-AP that utilizes a lipid acceptor, shows that an RxxR motif containing R153 aligns to a motif previously identified in CEPT1 as a determinant of acceptor specificity^20^. We suggest that the locations of the acceptor and donor sites are conserved across the entire CDP-AP family, regardless of the identity of the substrates.

A unique feature of the structure of *Rs*PIPS when compared with *Af*2299 and *Af*DIPP synthase is the presence of a hydrophobic crevice between JM1, TM2 and TM5 (Fig. 2 and Supplementary Fig. 1i), which in the structure of *Rs*PIPS-FL accommodates the lipid substrate (Fig. 3 and Supplementary Fig. 4). This groove is directly exposed to the bulk lipid, providing a pathway for lateral diffusion of CDP-DAG into the active site. The nucleotide is wedged between TM2 and TM3 in a pocket, which is also lined by TM1. The CDP interacts with residues from the signature sequence on TM2 and TM3. In addition, D29 and T32, part of a conserved P(D/N)xx(T/S) motif at the start of TM1, form hydrogen bonds with polar substituents of the pyrimidine ring. Given the absolute conservation of residues lining the nucleotide-binding pocket, we anticipate this mode of binding will be universally conserved.

PI is an essential lipid for mycobacteria, providing the anchor and first building block of major constituents of their cell wall^1^. Genetic ablation of PIP synthase in *Mycobacterium smegmatis* leads to a loss of cell viability^11^. This observation, combined with the unique substrate requirements of *Mt*PIPS, positions this enzyme as a plausible target for the development of novel anti-tuberculosis therapeutics. The structure of *Rs*PIPS provides a high-homology model for *Mt*PIPS (40% identity; Supplementary Fig. 5). We opted to perform experiments aimed at functional characterization of PIP synthase on *Mt*PIPS, due to its higher intrinsic medical interest and the low specific activity of the native *Rs*PIPS enzyme (Supplementary Fig. 1j). The conserved pocket that accommodates the inositol phosphate acceptor provides a potentially attractive site for future structure-based drug design for two reasons. First, inositol phosphate is not recognized by eukaryotic CDP-APs. Second, the affinity of *Mt*PIPS for inositol phosphate is relatively low (Fig. 4d), making displacement by a putative inhibitor more feasible, at least theoretically. Mutation of either of the two conserved arginine residues (R155 and R195) in this pocket resulted in gravely impaired enzymatic activity. We hypothesize that these two residues are responsible for binding of the phosphate of inositol phosphate. Interestingly, although mutation of R195 to a glutamine resulted in severely compromised activity and a increased *K*_M_ for inositol phosphate, the binding of inositol phosphate to liposome-incorporated R195Q was reduced only to a moderate degree (Fig. 4e), suggesting R195 may play an additional role in the reaction mechanism beyond its contribution to inositol phosphate affinity, potentially in positioning this substrate appropriately for catalysis. Mutation of residues that line the diacylglycerol-binding groove on either TM2 or TM5 to bulky tryptophans also compromised enzymatic activity of *Mt*PIPS, presumably by obstructing the groove that accommodates the acyl chains of CDP-DAG. We suggest that similar mutations in an ancestral lipid-processing CDP-AP may have contributed to the evolution of polar-osmolyte generating CDP-APs^10^, like *Af*DIPP synthase, providing a plausible explanation for the existence of an integral membrane enzyme that processes exclusively soluble products and substrates.

Finally, measurements of the binding of inositol phosphate to liposomes containing incorporated *Mt*PIPS-FL (WT and D93N) in the presence and absence of CDP-DAG showed that binding of inositol phosphate is strictly CDP-DAG dependent, and that the D93N mutation, while it almost completely abrogated activity of the enzyme, does not substantially impact substrate affinity (Fig. 4e). The observation that CDP-DAG binding is a prerequisite for inositol phosphate binding (and hence catalysis) implies that *Mt*PIPS follows a sequential ordered bi-bi reaction mechanism in which CDP-DAG binds first, followed by inositol phosphate, and the likely formation of a reactive phosphoryl intermediate through the action of an aspartate residue (D_4_ in the signature sequence) acting as a catalytic base, in this case D93. This is consistent with mechanisms described previously for several other members of the family^7^,^21^,^22^,^23^.

## Methods

### Target identification and cloning

CDP-alcohol phosphotransferases with predicted involvement in PIP synthesis were identified from 14 prokaryotic organisms by homology to a template of known function. Six mutations were introduced into each one (Supplementary Fig. 1) to replicate the interface between the cytosolic and TM domains observed in the structure of *Af*2299, and the corresponding genes were synthesized (GenScript). Genes not bearing the mutations at the interface were PCR amplified from the matching genomes. The Uniprot IDs and species of the sequences identified were as follows: 1: Q9F7Y9, *M. smegmatis*; 2: G6X547, *Mycobacterium abscessus*; 3: K0UMF3, *Mycobacterium fortuitum subsp. fortuitum*; 4: R4N892, *Mycobacterium avium subsp. paratuberculosis*; 5: D5MTP6, *Mycobacterium marinum*; 6: Q7D6W6, *M. tuberculosis*; 7: H6MZX4, *Gordonia polyisoprenivorans*; 8: Q0S1E0, *Rhodococcus sp.* (strain *RHA1*); 9: Q5YTD3, *Nocardia farcinica*; 10: D9UX52, *Streptomyces sp. AA4*; 11: F5XFI2, *Microlunatus phosphovorus*; 12: K9B2F1, *Brevibacterium casei*; 13: A9WSF5, *R. salmoninarum*; and 14: K1ENZ2, *Janibacter hoylei*. PCR was used to amplify the bacterial expression vector pMCSG7 encoding *Af*2299 (with an N-terminal decahistidine tag and a Tobacco Etch Virus (TEV) protease cleavage site), excluding the portion of the gene not encoding the N-terminal soluble domain. Gibson assembly^24^ was used to fuse the genes encoding PIP synthases to the linear fragment of the pMCSG7-*Af*2299 vector. All point mutants of *Mt*PIPS were generated using the QuikChange site-directed mutagenesis kit (Agilent). Sequences of all primers used for cloning and mutagenesis are provided in Supplementary Table 1.

### Membrane isolation and protein expression and purification

For protein overexpression, plasmids encoding PIP synthases, generated as described above, were transformed into BL21 (DE3) pLysS *E. coli* competent cells. Transformed cells were used to inoculate a starter culture (8 mL) of 2xYT medium supplemented with 100 μg ml^−1^ ampicillin and 50 μg ml^−1^ chloramphenicol. This culture was grown at 37 °C overnight while shaking (240 r.p.m.). The next day, the starter culture was used to inoculate 800 ml of 2xYT medium supplemented with 100 μg ml^−1^ ampicillin and 50 μg ml^−1^ chloramphenicol. Cultures were again grown at 37 °C while shaking (240 r.p.m.). Once the OD_600_ reached 1.0 (after about 3 h), the shaker temperature was reduced to 22 °C, and 15 min later protein expression was induced with a final concentration of 0.2 mM isopropyl β-D-1-thiogalactopyranoside. After overnight induction at 22 °C, cells were collected by centrifugation at 4,000*g* for 15 min at 4 °C and stored at −80 °C until needed. Cultures for large-scale protein expression were 800 ml in volume, while 15-ml cultures were grown similarly to test protein expression in small scale.

For large-scale purification of PIP synthases, frozen cell pellets were resuspended in lysis buffer containing 20 mM HEPES-NaOH (pH 7.5), 200 mM NaCl, 20 mM MgSO_4_, 10 μg ml^−1^ DNase I, 10 μg ml^−1^ RNase A, 1 mM TCEP, 1 mM phenylmethyl sulfonyl fluoride (PMSF) and Complete Mini EDTA-free protease inhibitor cocktail (Roche) used as described in the instructions. Cells were lysed with an Emulsiflex C3 homogenizer (Avestin). Lysate was solubilized for 1.5 h with 1% (w/v) *n*-dodecyl-β-D-maltopyranoside (DDM, Anagrade, Affymetrix) in a volume of ∼40 ml per cell pellet from 800 ml culture (∼6 g cells). Insoluble material was then pelleted by ultracentrifugation at 134,000*g* for 30 min at 4 °C. Protein was purified from the supernatant by immobilized metal-affinity chromatography (Ni-NTA, Qiagen). The soluble fraction was incubated with pre-equilibrated Ni-NTA beads (0.5 ml for 40 ml soluble fraction) for 2 h. The beads were washed with 10 column volumes of 20 mM HEPES-NaOH (pH 7.5), 200 mM NaCl, 0.1% (w/v) DDM and 60 mM imidazole (pH 7.5). The protein was then eluted from the beads with 5 column volumes of 20 mM HEPES-NaOH (pH 7.5), 200 mM NaCl, 0.05% (w/v) DDM and 300 mM imidazole (pH 7.5). Ni-NTA elutions were dialysed overnight in a Slide-A-Lyzer dialysis cassette (Thermo Scientific) at 4 °C in the presence of TEV protease (150 μl at 3 mg ml^−1^) to cleave the decahistidine tag. The dialysis buffer consisted of 20 mM HEPES-NaOH (pH 7.0), 200 mM NaCl and 0.05% (w/v) DDM. The next day, the sample was removed from the dialysis cassette and purified again using washed Ni-NTA to remove TEV protease, cleaved decahistidine tags and any non-cleaved protein. Flow-through containing purified cleaved protein was subjected to SEC using a Superose 12 column (GE Healthcare) in a buffer of 20 mM HEPES-NaOH (pH 7.0), 200 mM NaCl, 0.025% (w/v) DDM and 1 mM Tris(2-carboxyethyl)phosphine hydrochloride (TCEP-HCl). Protein eluted as a sharp monodisperse peak, as could be judged by monitoring A280. Approximately 0.75 mg of purified protein could be obtained from an 800-ml bacterial culture.

Small-scale initial protein expression tests were performed similarly using 100 mg quantities of cells from a 15-ml culture. Lysis was performed using a tip sonicator (3 × 5-s pulses with 5-s cooling intervals between pulses), and purification proceeded until the first immobilized metal-affinity chromatography step, after which the Ni-NTA elutions were mixed with 6 × SDS loading buffer and run on 12 or 14% SDS–PAGE gels to identify expressing PIP-synthase constructs.

For isolation of membranes, frozen cell pellets were resuspended in lysis buffer containing 20 mM HEPES-NaOH (pH 7.5), 200 mM NaCl, 20 mM MgSO_4_, 10 μg ml^−1^ DNase I, 10 μg ml^−1^ RNase A, 1 mM TCEP, 1 mM PMSF and Complete Mini EDTA-free protease inhibitor cocktail used as described in the instructions. Cells were lysed with an Emulsiflex C3 homogenizer (Avestin). The membrane fraction was pelleted by ultracentrifugation at 134,000*g* for 30 min at 4 °C. To remove water-soluble proteins, membranes were resuspended by homogenization in a high-salt buffer containing 20 mM HEPES-NaOH (pH 7.5), 500 mM NaCl, 20 mM MgSO_4_, 10 μg ml^−1^ DNase I, 10 μg ml^−1^ RNase A, 1 mM TCEP, 1 mM PMSF and Complete Mini EDTA-free protease inhibitor cocktail. The membrane fraction was pelleted once again by ultracentrifugation at 134,000*g* for 30 min at 4 °C. Membranes were then resuspended by homogenization in storage buffer containing 20 mM HEPES-NaOH (pH 7.5), 200 mM NaCl, 20 mM MgSO_4_ and 1 mM TCEP. If required, resuspended membranes were solubilized for 1.5 h with 1% (w/v) DDM. Protein purification was carried out as described above.

### Preparation of liposomes and proteoliposomes

*E. coli* polar lipid extract (Avanti) and phosphatdiylcholine (Avanti) were mixed in a 3:1 ratio (w/w) by dissolving in chloroform. Chloroform was removed under a stream of nitrogen gas to obtain a thin layer of dry lipids. Lipids were resuspended in buffer containing 100 mM HEPES, pH 7.5 and 1.5% (w/v) 1-*O*-*n*-Octyl-β-D-glucopyranoside (Anagrade, Affymetrix) and the detergent was removed by dialysis against 1 l of 100 mM HEPES, pH 7.5. The resulting liposomes were divided into aliquotes, frozen in liquid nitrogen and stored at −80 °C. For protein incorporation into liposomes, the protocol used was adapted from Rigaud *et al.*^25^. The concentration of thawed liposomes was adjusted to 10 mg ml^−1^ with 100 mM HEPES, pH 7.5; 0.11% (w/v) Triton X-100 was added to the liposome-containing solution and mixed by vortexing. Protein, purified as previously described, was then added in a ratio of 1:80 (0.125 mg protein to 10 mg lipid). The mixture was incubated at room temperature with agitation for 15 min. A unit of 60 mg of pretreated and equilibrated Bio-Beads SM-2 (BioRad) were added to the mixture and incubated at room temperature for 1 h under constant agitation. An additional 60 mg of Bio-Beads were then added to the mixture and incubated at room temperature for 1 h under constant agitation. Then, 120 mg of Bio-Beads were added to the mixture and incubated overnight at 4 °C with under constant agitation, after which proteoliposomes were separated and removed from the Bio-Beads by careful pipetting. The concentration of proteoliposomes was adjusted by ultracentrifugation (148,000*g* for 30 min at 4 °C) and resuspension in the correct volume of buffer (100 mM HEPES, pH 7.5). Proteoliposomes were divided into aliquotes, flash frozen in liquid nitrogen and stored at −80 °C.

### Preparation of cell-free homogenates for functional assays

Frozen recombinant cells of *E. coli* expressing PIP-synthase constructs (∼2 g), grown as described above, were suspended in 5 ml buffer A (50 mM Tris-HCl, pH 7.6, 10 mM MgCl_2_ and 5 mM β-mercaptoethanol) and disrupted by sonication (5 × 60-s pulses with 60-s cooling intervals between pulses). Cell debris and unbroken cells were separated by centrifugation (10,000*g*, 10 min, 4 °C), and membrane fractions were obtained by centrifugation of the supernatant at 100,000*g* for 2 h at 4 °C. The membrane fractions were suspended in 0.5 ml of buffer A and frozen at −20 °C until use.

### Preparation of L-*myo*-[^14^C]inositol-1-phosphate

L-*myo*-[^14^C]inositol-1-phosphate was prepared from [^14^C(U)]glucose (Perkin Elmer Life Sciences) using hexokinase of *Thermoproteus tenax* and L-*myo*-inositol-1-phosphate synthase (IPS) of *Archaeoglobus fulgidus*. *E. coli* cells harbouring the hexokinase or the *ips* genes were grown in Luria–Bertani medium at 37 °C supplemented with 100 μg ml^−1^ ampicillin to an optical density of 0.5 at 600 nm, and protein expression was induced for 4 h with 1 mM isopropyl β-D-1-thiogalactopyranoside^15^. Partial purification of recombinant hexokinase and IPS was performed by heating the cell extracts for 30 min at 90 and 60 °C, respectively, followed by centrifugation to remove denatured proteins. The production of [^14^C]glucose-6-phosphate was carried out in a reaction mixture containing the recombinant hexokinase, [^14^C(U)]glucose (3.7 MBq per 336 nmol), 10 mM glucose, 5 mM ATP, 50 mM Tris-HCl (pH 7.6) and 10 mM MgCl_2_. After 1 h of incubation at 70 °C, the reaction mixture was centrifuged (10,000*g*, 10 min, 4 °C), and the resulting supernatant was added to a reaction mixture containing the recombinant IPS, 5 mM NAD^+^ and 50 mM Tris-HCl (pH 7.6). After incubation at 85.5 °C for 1 h, and centrifugation (10,000*g*, 10 min, 4 °C), the resulting supernatant was treated with activated charcoal to eliminate residual nucleotides, and then filtered through a 10-kDa Omega Nanosep filter (Pall Life Sciences, Hampshire, UK) to remove proteins. The filtrate contained L-*myo*-[^14^C]inositol-1-phosphate, [^14^C]glucose-6-phosphate and [^14^C]glucose. L-*myo*-[^14^C]inositol-1-phosphate present in the preparation was quantified after thin-layer chromatography separation and used as a substrate for assays of PIP-synthase activity.

### Measurement of PIP-synthase activity

The reaction mixtures (final volume, 200 μl) contained the membrane fraction (200 μg of total membrane protein as determined by the Bradford method) of *E. coli* expressing PIPS constructs, 6.5 μM L-*myo*-[^14^C]inositol-1-phosphate, 161 μM of cold inositol phosphate prepared as described above, 0.4 mM CDP-dioleoylglycerol (Avanti Polar Lipids), 10 mM MgCl_2_, 10 mM β-mercaptoethanol, 1% (w/v) 3-[(3-cholamidopropyl)dimethylammonio]-1-propanesulfonate (CHAPS) and 50 mM Bicine buffer (pH 8.0). The reaction was started by the addition of the membrane fraction. The mixtures were incubated at 37 °C during 1 h and reactions were stopped by addition of 1 ml of 0.1 M HCl in methanol. The mixtures were transferred to glass tubes containing 1.5 ml 0.1 M HCl in methanol and 2.5 ml CHCl_3_. The partition into aqueous and organic layers was carried out with addition of 2.15 ml MgCl_2_ (1 M, pH 2). The organic layer was removed and washed twice with 0.1 M HCl, methanol/1 M MgCl_2_ (1:0.8, v/v). The radiolabelled product (in the organic layer) was quantified using a liquid scintillation counter (Beckman LS 6,500). To study the effect of 
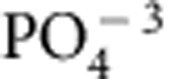
 and 
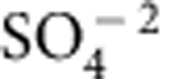
 on PIP-synthase activity, different concentrations of each compound (ranging from 1 to 200 mM, in the form of K_2_HPO_4_ and K_2_SO_4_) were added to the reaction mixture containing 200 μg of total membrane protein of *E. coli* expressing *Mt*PIPS-WT, 6.5 μM L-*myo*-[^14^C]inositol-1-phosphate, 161 μM of cold inositol phosphate, 0.4 mM CDP-dioleoylglycerol, 10 mM MgCl_2_, 10 mM β-mercaptoethanol, 1% (w/v) CHAPS and 50 mM Bicine buffer (pH 8.0). The reaction mixtures were incubated at 37 °C for 1 h and the extraction and quantification of the radiolabelled product was assessed as mentioned above. These functional assays were performed in triplicate.

### Assessment of *K*_M_ of *Mt*PIPS for inositol phosphate

The *K*_M_ of *Mt*PIPS (WT, R195Q and P153V) was assessed in reaction mixtures (final volume, 200 μl) containing 2 mM CDP-dioleoylglycerol, 10 mM MgCl_2_, 10 mM β-mercaptoethanol, 1% (w/v) CHAPS, 50 mM Bicine buffer (pH 8.0), 9 μM L-*myo*-[^14^C]inositol-1-phosphate plus different concentrations of cold inositol phosphate (final concentrations of 38 μM–1 mM for WT and P153V, and 38 μM–4 mM for R195Q).

The mixtures were pre-incubated for 3 min at 37 °C and the reactions initiated by addition of the membrane fraction of *E. coli* expressing *Mt*PIPS (200 μg of total membrane protein) and stopped at different time points by the addition of 1 ml of 0.1 M HCl in methanol. The extraction and quantification of the radiolabelled product was performed as described above. These experiments were performed in duplicate.

### Assessment of *K*_M_ of *Mt*PIPS for CDP-dioleoylglycerol

The *K*_M_ of *Mt*PIPS (WT, R195Q and P153V) for CDP-DAG was performed in reaction mixtures (final volume, 200 μl) containing 1 mM of inositol phosphate (from which 9 μM was L-*myo*-[^14^C]inositol-1-phospate), 10 mM MgCl_2_, 10 mM β-mercaptoethanol, 1% (w/v) CHAPS, 50 mM Bicine buffer (pH 8.0), plus different concentrations of CDP-dioleoylglycerol (ranging from 50 to 2,000 μM). The mixtures were pre-incubated for 3 min at 37 °C and the reactions initiated by the addition of the membrane fraction of *E. coli* expressing *Mt*PIPS (200 μg of protein) and stopped at different time points by addition of 1 ml of 0.1 M HCl in methanol. The extraction and quantification of the radiolabelled product was performed as described above. These experiments were performed in duplicate.

### Binding assays with L-*myo*-[^14^C]inositol-1-phosphate

L-*myo*-[^14^C]inositol-1-phosphate-binding assays were carried out in reaction mixtures (final volume, 100 μl) containing *Mt*PIPS (WT, D93N or R195Q) reconstituted in proteoliposomes (9 μg of protein), 2 mM MgCl_2_, 40 μM inositol phosphate (of which 16 μM was L-*myo*-[^14^C]inositol-1-phospate) and 50 mM Bicine buffer (pH 8.0). The mixtures were pre-incubated for 3 min at 37 °C absent ligand, the reactions initiated by the addition of inositol phosphate and stopped after 10 min. The binding assay mixtures were passed over HAWP 02500 filters (Millipore), and unbound L-*myo*-[^14^C]inositol-1-phosphate was separated from the bound by washing three times with 600 μl of 50 mM Bicine buffer (pH 8.0). Bound L-*myo*-[^14^C]inositol-1-phosphate was quantified by liquid scintillation counting. The effect of CDP-DAG on the binding of inositol phosphate to *Mt*PIPS was investigated by pre-incorporating 200 μM CDP-DAG in the proteoliposomes used in each reaction mixture. Assays and quantification of bound L-*myo*-[^14^C]inositol-1-phosphate were performed as described above. Assays on empty liposomes to calculate background were also performed as described above for proteoliposomes. These binding assays were performed in triplicate.

### Crystallization

Crystals were grown at room temperature (22 °C) in lipidic cubic phase, using as host lipid either monoolein alone (NuChek Prep) or a mixture of 2% CDP-dioleoylglycerol (Avanti Polar Lipids) and 98% monoolein by mass. The mixture of CDP-dioleoylglycerol and monoolein was prepared the day before it was needed, and involved dissolving CDP-dioleoylglycerol in chloroform, adding it to molten monoolein in the appropriate amount to generate a 2:98 ratio by mass, vortexing, and then evaporating the chloroform with argon gas first and then overnight in a vacuum desiccator. Protein from peak fractions from SEC was concentrated to 35–40 mg ml^−1^ (estimated by *A*_280nm_) for crystallization using a centrifugal concentrator (Millipore) with a 100 kDa molecular weight cutoff. Concentrated protein was mixed with molten lipid in a 1:1.5 (w/w) ratio of protein:lipid using coupled syringes. A Mosquito LCP (TTP Labtech) robot was used to dispense a typical volume of 50–75 nl of protein/lipid mixture onto a 96-well glass sandwich plate, which was covered with 750 nl precipitant solution and sealed with a glass coverslip. Glass sandwich plates were stored in a 22-°C incubator. Crystals appeared after 1–2 days and grew to full size in about 1 week. Crystals grew in (a) 20% (v/v) polyethylene glycol 400, 0.1 M MES (pH 6.7) and 0.2 M lithium sulfate (*Rs*PIPS-Δ6N), and (b) 30% (v/v) polyethylene glycol 300, 0.1 M MES pH 6.0, 0.1 M sodium chloride and 0.1 M magnesium chloride (*Rs*PIPS-FL in 2% CDP-DAG/98% monoolein). A tungsten carbide glass cutter (Hampton Research) was used to cut and remove the glass coverslip, and crystals were collected using 20–100 μm MicroLoops and MicroMounts (MiTeGen). Crystals were flash cooled directly in liquid nitrogen without additional cryoprotection. *Rs*PIPS-Δ6N crystallizes in space group P 2_1_ 2_1_ 2, with unit cell parameters (Å) *a*=48.63, *b*=94.07, *c*=103.92, with one protomer in the asymmetric unit, and diffraction to 2.5 Å. *Rs*PIPS-FL crystallizes in space group P 2_1_, with unit cell parameters (Å) *a*=89.00, *b*=62.49, *c*=169.76, *β*=99.77^o^, with two dimers in the asymmetric unit, and diffraction to 3.6 Å.

### Data collection and structure determination

Diffraction data were collected on beamlines 24-ID-C and 24-ID-E at the Advanced Photon Source (Argonne, IL). The data were indexed, integrated, scaled and merged using XDS^26^ and AIMLESS^27^. The structure of *Rs*PIPS-Δ6N was solved by molecular replacement using PHASER^28^, searching separately for the extramembrane and transmembrane domains of *Af*2299 (PDB ID 4O6M). The final data set includes data collected from four isomorphous crystals. After density modification using PARROT^29^, the model was manually corrected and completed using Coot^30^, and refined using the PHENIX crystallographic software package^31^, alternating between cycles of manual building in Coot and refinement in PHENIX. The final *Rs*PIPS-Δ6N model has an *R*_work_/*R*_free_ of 0.2284/0.2520. The structure of *Rs*PIPS-FL was solved by molecular replacement using PHASER, searching separately for four copies each of the extramembrane and transmembrane domains from the structure of *Rs*PIPS-Δ6N. Density modification, including non-crystallographic averaging, was performed using PARROT, and the model was completed following the same protocol as for the structure of *Rs*PIPS-Δ6N, using the structure of *Rs*PIPS-Δ6N as a reference model for the generation of restraints 32, in addition to the application of non-crystallographic symmetry based torsion angle restraints and secondary structure restraints, giving a model with a final *R*_work_/*R*_free_ of 0.2801/0.2997. All protein structure figures were prepared using UCSF Chimera^33^. In the structure of *Rs*PIPS-Δ6N, many partially ordered lipid molecules were readily apparent in the electron density map (Supplementary Fig. 8). As no head groups or identifying features were discernable in the density, all lipids were modelled as isolated alkyl chains and assigned the residue code UNL, the PDB-recommended code for all unidentified ligands.

## Additional information

**Accession codes:** Coordinates and structure factors have been deposited in the Protein Data Bank under the accession codes 5D91 (RsPIPS-Δ6N) and 5D92 (RsPIPS-FL).

**How to cite this article:** Clarke, O. B. *et al.* Structural basis for phosphatidylinositol-phosphate biosynthesis. *Nat. Commun.* 6:8505 doi: 10.1038/ncomms9505 (2015).

## Supplementary Material

Supplementary Information Supplementary Figures 1-8, Supplementary Table 1 and Supplementary References

Supplementary Movie 1 "Transmembrane architecture and CDP-DAG binding site of PIP-synthase from Renibacterium salmoninarum

## Figures and Tables

**Table 1:** Data collection and refinement statistics.

	***Rs*****PIPS-Δ6N**	***Rs*****PIPS-FL**
Wavelength (Å)	0.979	0.979
Resolution range collected (Å)	103.92–2.50 (2.60–2.50)	167.3–3.613 (3.97–3.61)
Resolution range used in refinement(Å)	14.92–2.502 (2.591–2.502)	167.3–3.613 (3.742–3.613)
Space group	P 2_1_ 2_1_ 2	P 2_1_
Unit cell	*a*=48.63	*a=*89.00
	*b=*94.069	*b=*62.49
	*c=*103.92	*c=*169.76
		*β=*99.77
Total reflections	320,975 (21,993)	78,622 (18,367)
Unique reflections	16,784 (1,453)	21,268 (1,996)
Multiplicity	19.0 (13.6)	3.7 (3.8)
Completeness (%)	98.2 (84.5)	98.9 (97.9)
Mean *I*/sigma(*I*)	9.9 (1.7)	5.0 (1.4)
Wilson *B*-factor	43.74	79.46
*R*_merge_	0.267 (1.654)	0.252 (0.930)
*R*_meas_	0.281 (1.775)	0.294 (1.087)
CC(1/2)	0.999 (0.636)	0.991 (0.616)
Resolution where *I*/sigma(*I*) drops below 2.0 (overall)	2.59	3.89
Resolution where *I*/sigma(*I*) drops below 2.0 (along *h*)	2.50	4.09
Resolution where *I*/sigma(*I*) drops below 2.0 (along *k*)	3.25	4.09
Resolution where *I*/sigma(*I*) drops below 2.0 (along *l*)	2.61	3.62
Resolution where CC(1/2) drops below 0.5 (overall)	2.50	3.62
Resolution where CC(1/2) drops below 0.5 (along *h*)	2.50	3.84
Resolution where CC(1/2) drops below 0.5 (along *k*)	3.34	4.05
Resolution where CC(1/2) drops below 0.5 (along *l*)	2.59	3.62
Reflections used in refinement	16,891 (1,453)	21,268 (1,986)
Reflections used for *R*_free_	824 (58)	1,043 (117)
*R*_work_	0.2284 (0.3326)	0.2801 (0.3918)
*R*_free_	0.2520 (0.3627)	0.2997 (0.4246)
Number of atoms (all non-hydrogen)	2,952	10,845
Number of atoms (protein)	2,586	10,350
Number of atoms (ligands)	333	495
Protein residues	338	1,358
RMS (bonds)	0.003	0.004
RMS (angles)	0.72	1.07
Ramachandran favoured (%)	98	97
Ramachandran allowed (%)	2	1.9
Ramachandran outliers (%)	0	0.1
Rotamer outliers (%)	1.8	1.7
Clashscore	2.87	8.53
Average *B*-factor (all atoms)	70.55	76.53
Average *B*-factor (protein)	70.89	77.17
Average *B*-factor (ligands)	69.59	63.13
Average *B*-factor (solvent)	53.66	
Number of TLS groups	2	

Statistics for the highest-resolution shell are shown in parentheses; RMS, root mean square; TLS, Translation-Libration-Screw.

## References

[b1] Umesiri F. E., Sanki A. K., Boucau J., Ronning D. R. & Sucheck S. J. Recent advances toward the inhibition of mAG and LAM synthesis in Mycobacterium tuberculosis. Med. Res. Rev. 30, 290–326 (2010). 20099253 10.1002/med.20190

[b2] Angala S. K., Belardinelli J. M., Huc-Claustre E., Wheat W. H. & Jackson M. The cell envelope glycoconjugates of Mycobacterium tuberculosis. Crit. Rev. Biochem. Mol. Biol. 49, 1–39 (2014). 24915502 10.3109/10409238.2014.925420 PMC4436706

[b3] Fischl A. S. & Carman G. M. Phosphatidylinositol biosynthesis in Saccharomyces cerevisiae: purification and properties of microsome-associated phosphatidylinositol synthase. J. Bacteriol. 154, 304–311 (1983). 6300035 10.1128/jb.154.1.304-311.1983 PMC217460

[b4] Morii H., Ogawa M., Fukuda K., Taniguchi H. & Koga Y. A revised biosynthetic pathway for phosphatidylinositol in Mycobacteria. J. Biochem. 148, 593–602 (2010). 20798167 10.1093/jb/mvq093

[b5] Morii H., Kiyonari S., Ishino Y. & Koga Y. A novel biosynthetic pathway of archaetidyl-myo-inositol via archaetidyl-myo-inositol phosphate from CDP-archaeol and D-glucose 6-phosphate in methanoarchaeon Methanothermobacter thermautotrophicus cells. J. Biol. Chem. 284, 30766–30774 (2009). 19740749 10.1074/jbc.M109.034652 PMC2781475

[b6] Carman G. M. & Belunis C. J. Phosphatidylglycerophosphate synthase activity in Saccharomyces cerevisiae. Can. J. Microbiol. 29, 1452–1457 (1983). 6318941 10.1139/m83-222

[b7] Bae-Lee M. S. & Carman G. M. Phosphatidylserine synthesis in Saccharomyces cerevisiae. Purification and characterization of membrane-associated phosphatidylserine synthase. J. Biol. Chem. 259, 10857–10862 (1984). 6088519

[b8] Sciara G. *et al.* Structural basis for catalysis in a CDP-alcohol phosphotransferase. Nat. Commun. 5, 4068 (2014). 24923293 10.1038/ncomms5068 PMC4098843

[b9] Nogly P. *et al.* X-ray structure of a CDP-alcohol phosphatidyltransferase membrane enzyme and insights into its catalytic mechanism. Nat. Commun. 5, 4169 (2014). 24942835 10.1038/ncomms5169

[b10] Gonçalves L. G. *et al.* Evolution of the biosynthesis of di-myo-inositol phosphate, a marker of adaptation to hot marine environments. Environ. Microbiol. 14, 691–701 (2011). 22026421 10.1111/j.1462-2920.2011.02621.x

[b11] Jackson M., Crick D. C. & Brennan P. J. Phosphatidylinositol is an essential phospholipid of mycobacteria. J. Biol. Chem. 275, 30092–30099 (2000). 10889206 10.1074/jbc.M004658200

[b12] Salman M., Lonsdale J. T., Besra G. S. & Brennan P. J. Phosphatidylinositol synthesis in mycobacteria. Biochim. Biophys. Acta 1436, 437–450 (1999). 9989274 10.1016/s0005-2760(98)00151-9

[b13] Morii H. *et al.* Studies of inositol 1-phosphate analogues as inhibitors of the phosphatidylinositol phosphate synthase in mycobacteria. J. Biochem. 153, 257–266 (2013). 23225597 10.1093/jb/mvs141

[b14] Brito J. A., Borges N., Vonrhein C., Santos H. & Archer M. Crystal structure of Archaeoglobus fulgidus CTP:inositol-1-phosphate cytidylyltransferase, a key enzyme for di-myo-inositol-phosphate synthesis in (hyper)thermophiles. J. Bacteriol. 193, 2177–2185 (2011). 21378188 10.1128/JB.01543-10 PMC3133074

[b15] Rodrigues M. V. *et al.* Bifunctional CTP:inositol-1-phosphate cytidylyltransferase/CDP-inositol:inositol-1-phosphate transferase, the key enzyme for di-myo-inositol-phosphate synthesis in several (hyper)thermophiles. J. Bacteriol. 189, 5405–5412 (2007). 17526717 10.1128/JB.00465-07 PMC1951816

[b16] Caffrey M. Crystallizing membrane proteins for structure determination: use of lipidic mesophases. Annu. Rev. Biophys. 38, 29–51 (2009). 19086821 10.1146/annurev.biophys.050708.133655

[b17] Fryer J. L. & Sanders J. E. Bacterial kidney disease of salmonid fish. Annu. Rev. Microbiol. 35, 273–298 (1981). 6794423 10.1146/annurev.mi.35.100181.001421

[b18] Williams J. G. & McMaster C. R. Scanning alanine mutagenesis of the CDP-alcohol phosphotransferase motif of Saccharomyces cerevisiae cholinephosphotransferase. J. Biol. Chem. 273, 13482–13487 (1998). 9593682 10.1074/jbc.273.22.13482

[b19] Chun E. *et al.* Fusion partner toolchest for the stabilization and crystallization of G protein-coupled receptors. Structure 20, 967–976 (2012). 22681902 10.1016/j.str.2012.04.010 PMC3375611

[b20] Henneberry A. L., Wright M. M. & McMaster C. R. The major sites of cellular phospholipid synthesis and molecular determinants of fatty acid and lipid head group specificity. Mol. Biol. Cell 13, 3148–3161 (2002). 12221122 10.1091/mbc.01-11-0540 PMC124149

[b21] Dutt A. & Dowhan W. Purification and characterization of a membrane-associated phosphatidylserine synthase from Bacillus licheniformis. Biochemistry 24, 1073–1079 (1985). 3006742 10.1021/bi00326a001

[b22] Hirabayashi T., Larson T. J. & Dowhan W. Membrane-associated phosphatidylglycerophosphate synthetase from Escherichia coli: purification by substrate affinity chromatography on cytidine 5'-diphospho-1,2-diacyl-sn-glycerol sepharose. Biochemistry 15, 5205–5211 (1976). 793612 10.1021/bi00669a002

[b23] Aktas M. *et al.* Enzymatic properties and substrate specificity of a bacterial phosphatidylcholine synthase. FEBS J. 281, 3523–3541 (2014). 24931117 10.1111/febs.12877

[b24] Gibson D. G. *et al.* Enzymatic assembly of DNA molecules up to several hundred kilobases. Nat. Methods 6, 343–345 (2009). 19363495 10.1038/nmeth.1318

[b25] Rigaud J. L., Pitard B. & Levy D. Reconstitution of membrane proteins into liposomes: application to energy-transducing membrane proteins. Biochim. Biophys. Acta 1231, 223–246 (1995). 7578213 10.1016/0005-2728(95)00091-v

[b26] Kabsch W. XDS. Acta Crystallogr. D Biol. Crystallogr. 66, 125–132 (2010). 20124692 10.1107/S0907444909047337 PMC2815665

[b27] Evans P. R. & Murshudov G. N. How good are my data and what is the resolution? Acta Crystallogr. D Biol. Crystallogr. 69, 1204–1214 (2013). 23793146 10.1107/S0907444913000061 PMC3689523

[b28] Mccoy A. J. *et al.* Phaser crystallographic software. J. Appl. Crystallogr. 40, 658–674 (2007). 19461840 10.1107/S0021889807021206 PMC2483472

[b29] Cowtan K. Recent developments in classical density modification. Acta Crystallogr. D Biol. Crystallogr. 66, 470–478 (2010). 20383000 10.1107/S090744490903947X PMC2852311

[b30] Emsley P., Lohkamp B., Scott W. G. & Cowtan K. Features and development of Coot. Acta Crystallogr. D Biol. Crystallogr. 66, 486–501 (2010). 20383002 10.1107/S0907444910007493 PMC2852313

[b31] Adams P. D. *et al.* PHENIX: a comprehensive Python-based system for macromolecular structure solution. Acta Crystallogr. D Biol. Crystallogr. 66, 213–221 (2010). 20124702 10.1107/S0907444909052925 PMC2815670

[b32] Headd J. J. *et al.* Use of knowledge-based restraints in phenix.refine to improve macromolecular refinement at low resolution. Acta Crystallogr. D Biol. Crystallogr. 68, 381–390 (2012). 22505258 10.1107/S0907444911047834 PMC3322597

[b33] Pettersen E. F. *et al.* UCSF Chimera—a visualization system for exploratory research and analysis. J. Comput. Chem. 25, 1605–1612 (2004). 15264254 10.1002/jcc.20084

[b34] Kyte J. & Doolittle R. F. A simple method for displaying the hydropathic character of a protein. J. Mol. Biol. 157, 105–132 (1982). 7108955 10.1016/0022-2836(82)90515-0

[b35] Voss N. R. & Gerstein M. 3V: cavity, channel and cleft volume calculator and extractor. Nucleic Acids Res. 38, W555–W562 (2010). 20478824 10.1093/nar/gkq395 PMC2896178

[b36] Kelley L. A. & Sternberg M. J. E. Protein structure prediction on the Web: a case study using the Phyre server. Nat. Protoc. 4, 363–371 (2009). 19247286 10.1038/nprot.2009.2

