# Supplementary material for: Structural basis for phosphatidylinositol-phosphate biosynthesis
Source: Nat Commun. 2015 Oct 16;0:8505. doi: 10.1038/ncomms9505 (PMC4634129; doi:10.1038/ncomms9505)
Supplement: Supplementary Information — Supplementary Figures 1-8, Supplementary Table 1 and Supplementary References [file ncomms9505-s1.pdf]

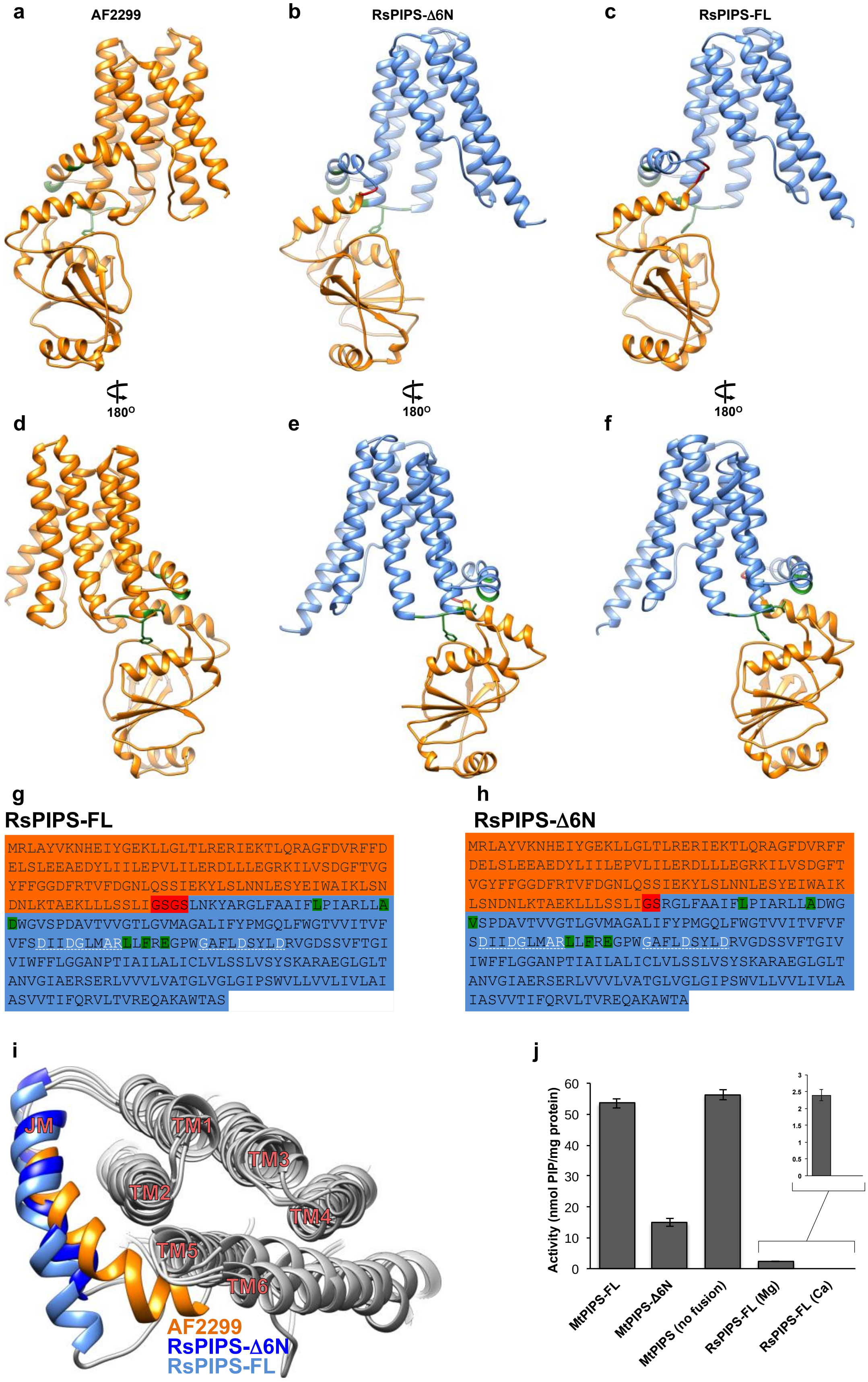
a
AF2299
b
RsPIPS-Δ6N
c
RsPIPS-FL
180°
180°
180°
d
e
f
g
RsPIPS-FL
MRLAYVKNHEIYGEKLLGLTLRERIEKTLQRAGFDVRFFD
ELSLEEAEDYLIIILEPVLILERDLLLEGRKILVSDGFTVG
YFFGGDFRTVFDGNLQSSIEKYLSLNNLESYEIWAIKLSN
DNLKTAEKLLLSSLIGSGSLNKYARGLFAAIFLPIARLLA
DWGVSPDAVTVVGTLGVMAGALIFYPMGQLFWGTVVITVF
VFSDIIDGLMARLLFREGPWGAFLDSYLDRVGDSSVFTGI
VIWFFLGGANPTIAILALICLVLSSLVSYSKARAEGLGLT
ANVGIAERSERLVVVLVATGLVGLGIPSWVLLVVLIVLAI
ASVVTIFQRVLTVREQAKAWTAS
h
RsPIPS-Δ6N
MRLAYVKNHEIYGEKLLGLTLRERIEKTLQRAGFDVRFF
DELSLEEAEDYLIIILEPVLILERDLLLEGRKILVSDGFT
VGYFFGGDFRTVFDGNLQSSIEKYLSLNNLESYEIWAIK
LSNDNLKTAEKLLLSSLIGSRGLFAAIFLPIARLLADWG
VSPDAVTVVGTLGVMAGALIFYPMGQLFWGTVVITVFVF
SDIIDGLMARLLFREGPWGAFLDSYLDRVGDSSVFTGIV
IWFFLGGANPTIAILALICLVLSSLVSYSKARAEGLGLT
ANVGIAERSERLVVVLVATGLVGLGIPSWVLLVVLIVLA
IASVVTIFQRVLTVREQAKAWTA
i
JM
TM1
TM2
TM3
TM4
TM5
TM6
AF2299
RsPIPS-Δ6N
RsPIPS-FL
j
Activity (nmol PIP/mg protein)
0
10
20
30
40
50
60
0
0.5
1
1.5
2
2.5
3
MtPIPS-FL
MtPIPS-Δ6N
MtPIPS (no fusion)
RsPIPS-FL (Mg)
RsPIPS-FL (Ca)

Supplementary Fig. 1 | Design of *Af*2299-*Rs*PIPS fusion constructs. Ribbon representation of *Af*2299 **(a, d)**, *Rs*PIPS-Δ6N **(b, e)**, and *Rs*PIPS-FL **(c, f)**. Sequence derived from *Af*2299 is colored orange, with the exception of residues that were mutated to replicate the TM-extramembrane domain interface observed in the structure of *Af*2299 (green; two residues, F77 and L75 have side chains depicted in stick representation). *Rs*PIPS sequence is colored in light blue, and the linker is colored red. The sequences of the *Rs*PIPS-FL **(g)** and *Rs*PIPS-Δ6N **(h)** constructs are colored as in panels A-F, with the addition that the CDP-AP signature sequence is highlighted and underlined in white. In *Af*2299, the juxtamembrane helix (orange) wraps around the TM domain (gray), forming a hydrophilic pocket that accommodates the polar CDP-glycerol donor, whereas in *Rs*PIPS-FL (light blue) and *Rs*PIPS-Δ6N (dark blue) it separates from the TM domain, forming a hydrophobic groove that shields the acyl chains of the CDP-diacylglycerol donor **(i)**. *Mt*PIPSΔ6N, which possesses a truncated juxtamembrane helix has compromised activity when compared with *Mt*PIPS-FL; *Mt*PIPS-FL has comparable activity to *Mt*PIPS (no fusion), the protein without the fusion and without the interface mutations; *Rs*PIPS-FL has significantly lower activity than the equivalent construct of the *Mycobacterium tuberculosis* protein (**j**).

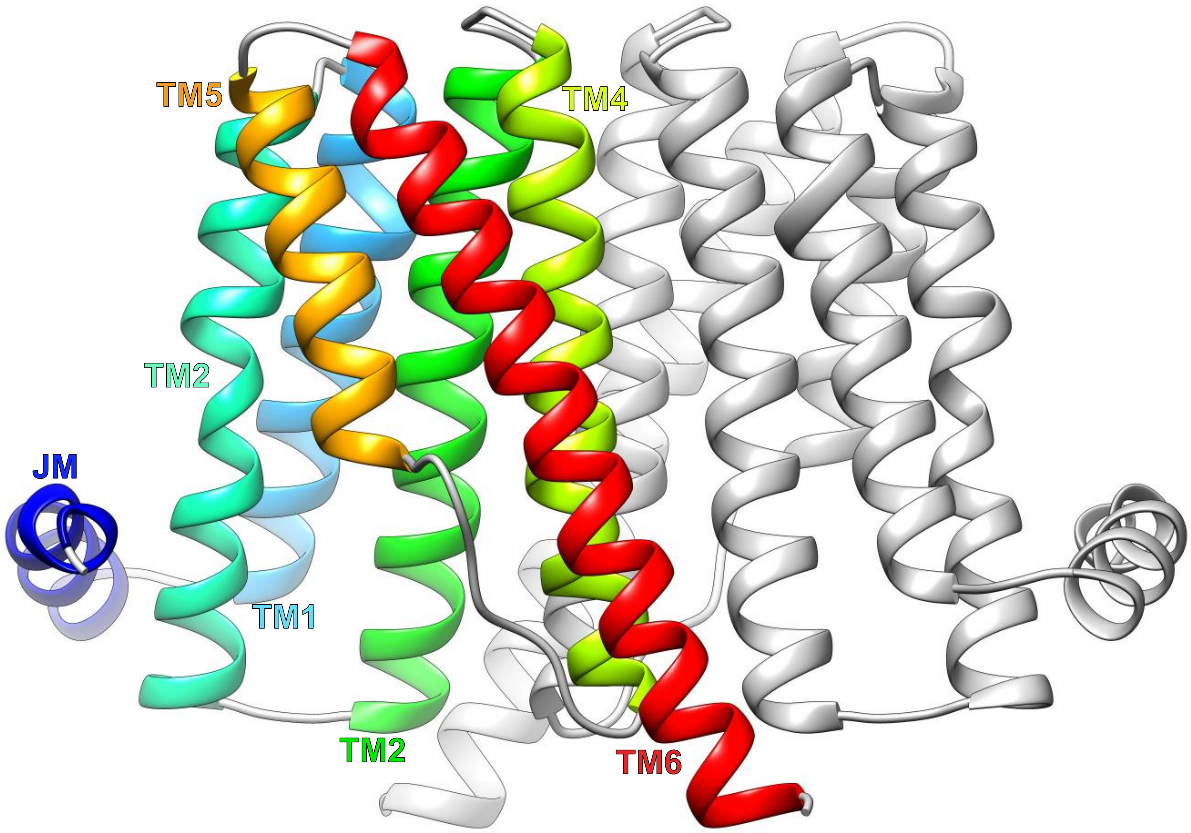

LNKYARGLFAAIFLPIARLLADWGV
SPDAVTVVGTLGVMAGALIFYPMGQ
LFWGTVVITVFVFSDIIDGLMARLL
FREGPWGAFLDSYLDRVGDSSVFTG
IVIWFFLGGANPTIAILALICLVLS
SLVSYSKARAEGLGLTANVGIAERS
ERLVVVLVATGLVGLGIPSWVLLVV
LIVLAIASVVTIFQRVLTVREQAKA
WTAS

Supplementary Fig. 2 | Transmembrane architecture of *Rs*PIPS. The transmembrane architecture of *Rs*PIPS-FL (upper panel) is shown in ribbon representation, colored from blue (JM1) to red (TM6). The sequence of *Rs*PIPS, with matching coloring, is shown in the lower panel.

a

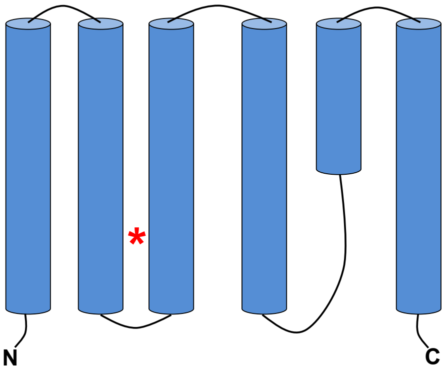

6TM: PgsA, eukaryotic cardiolipin synthases, PI synthase

b

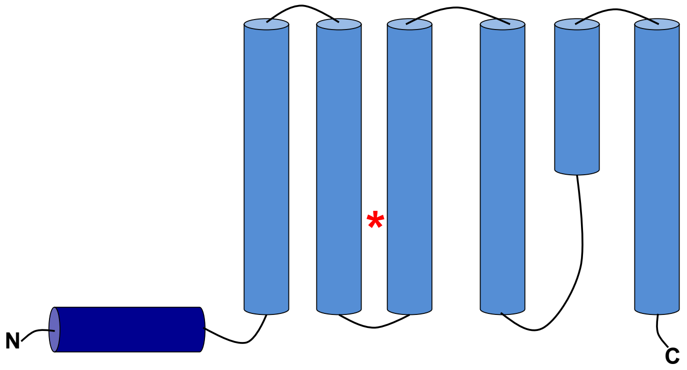

6TM+JM1: PIP synthases, AIP synthases, DIPP synthases, bacterial PC/PE synthases.

c

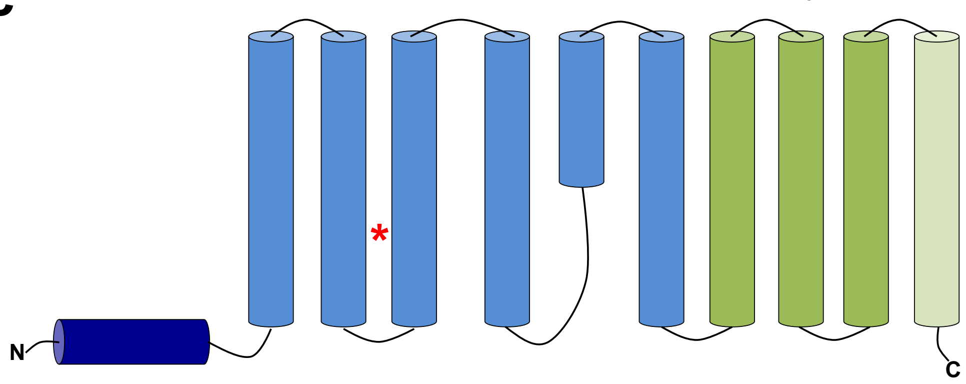

9/10TM+JM1: Eukaryotic PC/PE synthases

d

```
                                                              JM
RsPIPS   ---------------------------------------------MLNKYARGLFAAIFM  15
HsCEPT1  MSGHRSTRKRCGDSHPESPVGFGHMSTTGCVLNKLFQLPTPPLSRHQLKRLEEHRYQSAG  60

                                            1
RsPIPS   PIARLLVRWGVS------------PDAVTVVGTLGVMAGALIFYPMG----------QLF  53
HsCEPT1  RSLLEPLMQGYWEWLVRRVPSWIAPNLITIIGLSINICTTILLVFYCPTATEQAPLWAYI  120

                   2                                3
RsPIPS   WGTVVITVFVFSDIIDGLMARQLDRPGPWGAFLDSYLDRVGDSSVFTGIVIWFFLGGANP  113
HsCEPT1  ACACGLFIYQSLDAIDGKQARRTNSSSPLGELFDHGCDSLSTVFVVLGTCIAVQLGTNPD  180

                      4                                 5
RsPIPS   TIAILALICLVLSSLVSYSKARAEGLGLTANVGIAERSERLVVVLVATGLVGLGIP-SWV  172
HsCEPT1  WMFFCCFAGTFMFYCAH----WQTYVSGTLRFGIIDVTEVQIFIIIMHLLAVIGGPPFWQ  236

                        6
RsPIPS   LLVVLIVLAIASVVTIFQRVLTVREQAKAWTAS---------------------------  205
HsCEPT1  SMIPVLNIQMKIFPALCTVAGTIFSCTNYFRVIFTGGVGKNGSTIAGTSVLSPFLHIGSV  296
                                                               7

RsPIPS   ------------------------------------------------------------  205
HsCEPT1  ITLAAMIYKKSAVQLFEKHPCLYILTFGFVSAKITNKLVVAHMTKSEMHLHDTAFIGPAL  356
                                        8

RsPIPS   ------------------------------------------------------------  205
HsCEPT1  LFLDQYFNSFIDEYIVLWIALVFSFFDLIRYCVSVCNQIASHLHIHVFRIKVSTAHSNHH  416
                               9
```

Supplementary Fig. 3 | Transmembrane architecture of different groups of integral membrane CDP-APs with varying numbers of transmembrane helices. Some CDP-APs, **(a)**, like PgsA, eukaryotic cardiolipin synthases and eukaryotic PI-synthases, are expected to possess six transmembrane helices but lack the N-terminal juxtamembrane helices, while the PIP-synthase, AIP-synthase, and bacterial PC/PE synthases **(b)** are predicted to adopt the same architecture as *Rs*PIPS, with six transmembrane helices and a single N-terminal juxtamembrane helix. Eukaryotic CDP-APs that process a lipid acceptor, such as the eukaryotic PC/PE synthases **(c)**, are predicted to adopt an architecture consisting of the same core architecture as *Rs*PIPS, with six transmembrane helices (light blue) and one juxtamembrane helix (dark blue), with an additional 3 or 4 transmembrane helices (green). In panels A-C, the location of the CDP-AP signature sequence between TM2 and TM3 is represented by a red asterisk. **(d)** A sequence alignment of *Rs*PIPS (generated using PROMALS3D[1] followed by manual editing) with human choline/ethanolamine phosphotransferase (HsCEPT1), with the extra three transmembrane helices, as predicted by TMHMM2.0[2] highlighted in green, and the borders of the transmembrane helices observed in the *Rs*PIPS structure delineated in blue.

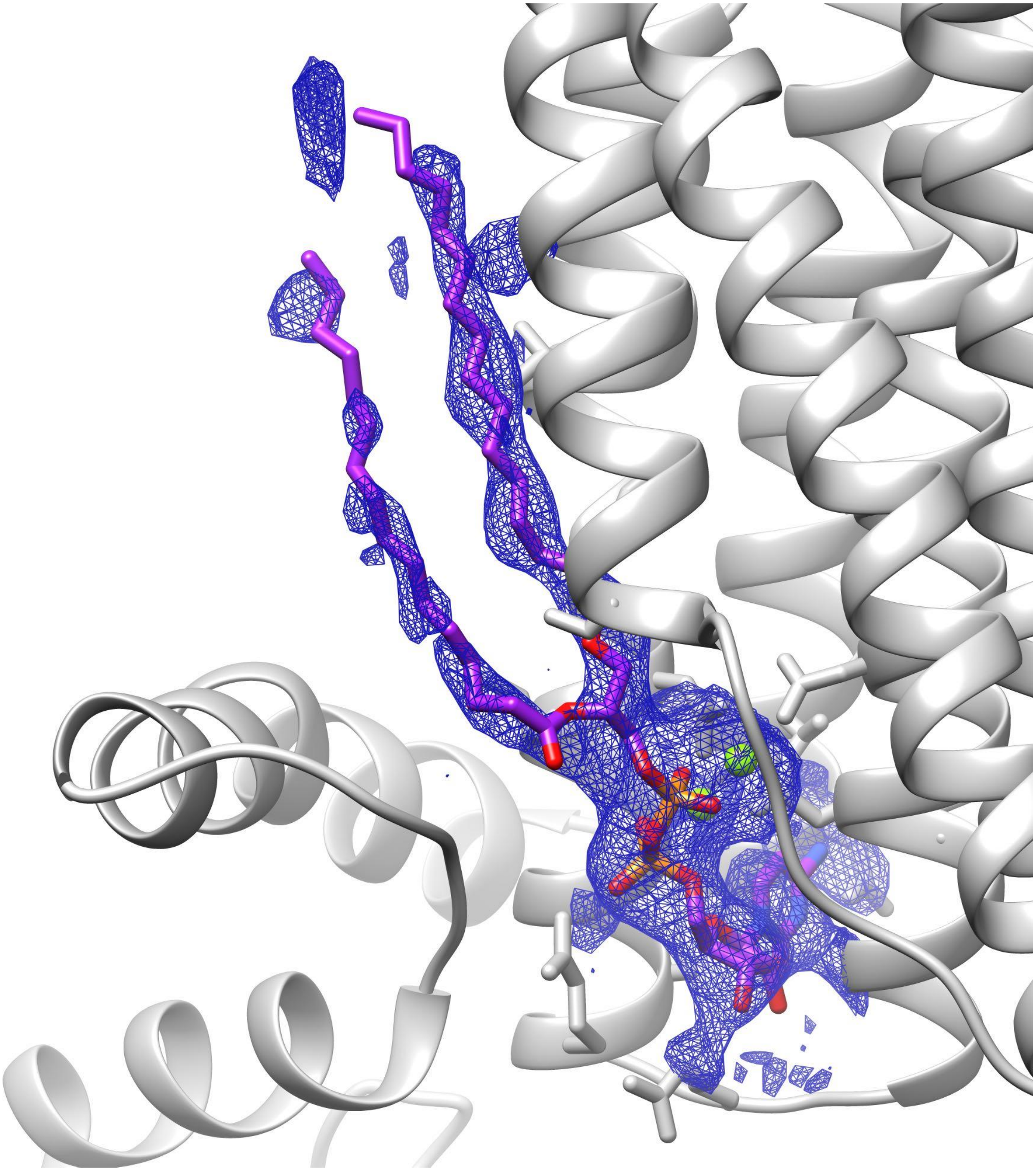

Supplementary Fig. 4 | CDP-DAG binding to *Rs*PIPS-FL. The structure of the *Rs*PIPS-FL CDP-DAG complex (protein in gray ribbons; CDP-DAG in purple stick representation) is shown with a $2mF_o$-DF_c map calculated prior to placement of the ligand (blue mesh, contoured at 0.8xR.M.S and shown within 4 Å of the ligand).

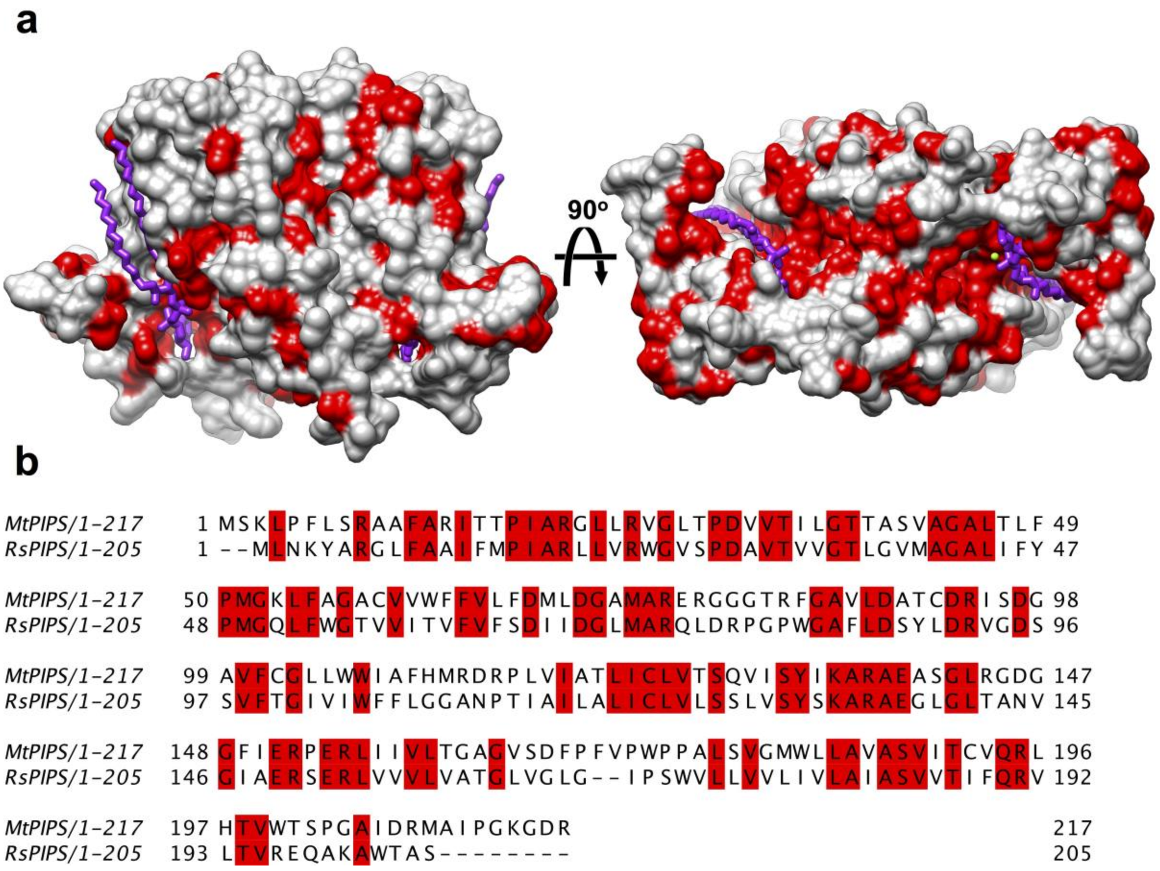

Supplementary Fig. 5 | Homology of *Rs*PIPS to *Mt*PIPS. (a) The structure of the transmembrane domain of *Rs*PIPS-FL, shown in spacefill representation, is colored in gray, with those residues that are identical in *Mt*PIPS colored red. CDP-DAG is shown in purple stick representation. **(b)** An alignment of *Mt*PIPS and *Rs*PIPS (generated using PROMALS3D[3]), with identical residues highlighted in red.

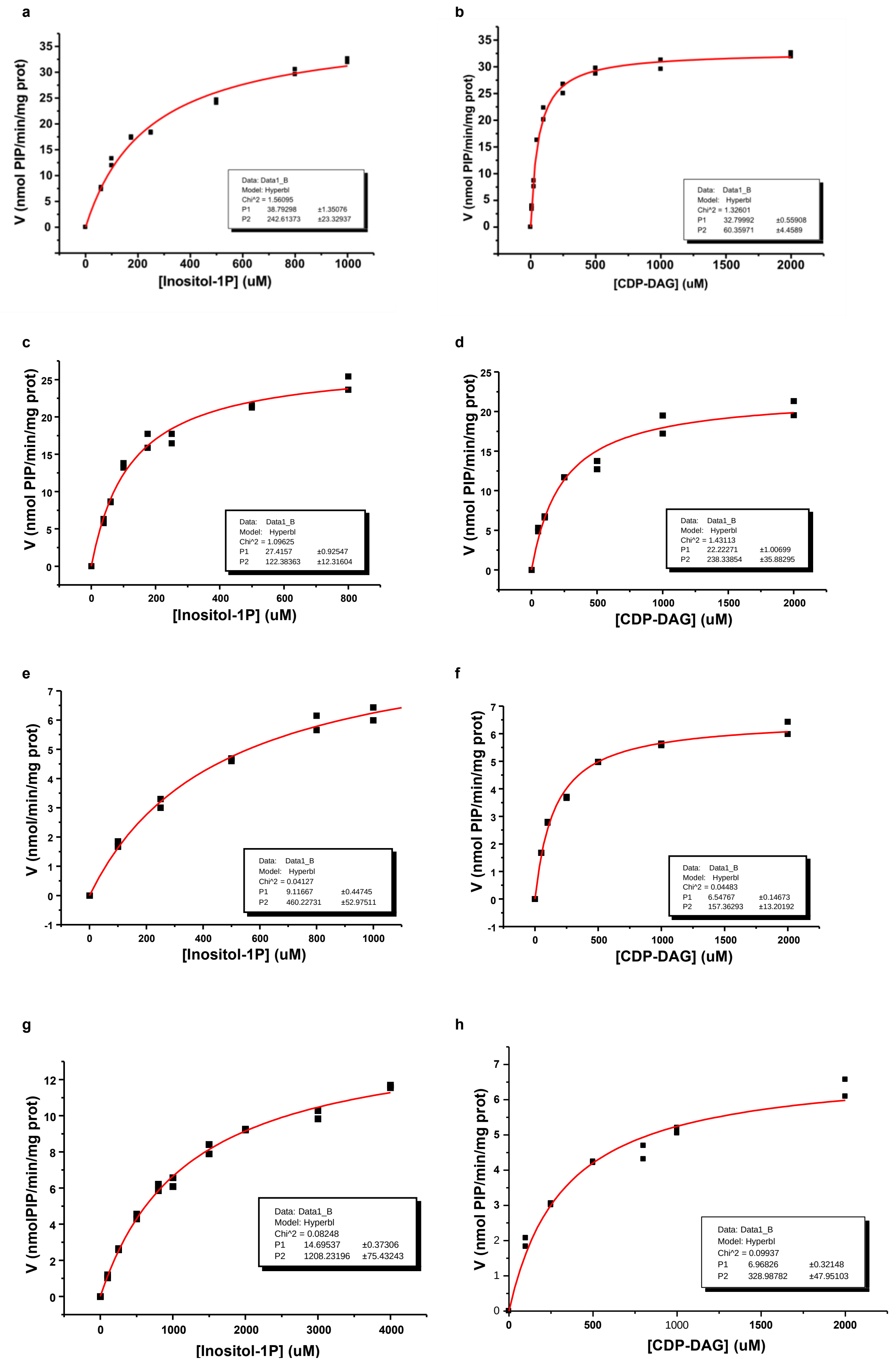
a
V (nmol PIP/min/mg prot)
[Inositol-1P] (uM)
Data: Data1_B
Model: Hyperbl
Chi^2 = 1.56095
P1 38.79298 ±1.35076
P2 242.61373 ±23.32937
b
V (nmol PIP/min/mg prot)
[CDP-DAG] (uM)
Data: Data1_B
Model: Hyperbl
Chi^2 = 1.32601
P1 32.79992 ±0.55908
P2 60.35971 ±4.4589
c
V (nmol PIP/min/mg prot)
[Inositol-1P] (uM)
Data: Data1_B
Model: Hyperbl
Chi^2 = 1.09625
P1 27.4157 ±0.92547
P2 122.38363 ±12.31604
d
V (nmol PIP/min/mg prot)
[CDP-DAG] (uM)
Data: Data1_B
Model: Hyperbl
Chi^2 = 1.43113
P1 22.22271 ±1.00699
P2 238.33854 ±35.88295
e
V (nmol/min/mg prot)
[Inositol-1P] (uM)
Data: Data1_B
Model: Hyperbl
Chi^2 = 0.04127
P1 9.11667 ±0.44745
P2 460.22731 ±52.97511
f
V (nmol PIP/min/mg prot)
[CDP-DAG] (uM)
Data: Data1_B
Model: Hyperbl
Chi^2 = 0.04483
P1 6.54767 ±0.14673
P2 157.36293 ±13.20192
g
V (nmolPIP/min/mg prot)
[Inositol-1P] (uM)
Data: Data1_B
Model: Hyperbl
Chi^2 = 0.08248
P1 14.69537 ±0.37306
P2 1208.23196 ±75.43243
h
V (nmol PIP/min/mg prot)
[CDP-DAG] (uM)
Data: Data1_B
Model: Hyperbl
Chi^2 = 0.09937
P1 6.96826 ±0.32148
P2 328.98782 ±47.95103

Supplementary Figure 6 | K_M of *Mt*PIPS (WT, P153V and R195Q) for inositol phosphate and CDP-DAG. Measurement of the activity of *Mt*PIPS-FL WT (No *Af*2299 fusion or interface mutations) **(a, b)**, *Mt*PIPS-FL WT (with *Af*2299 fusion and interface mutations) **(c, d)**, P153V **(e, f)**, and R195Q **(g, h)** in the presence of varying concentrations of either inositol phosphate (Inositol-1P) **(a, c, e, g)** or CDP-DAG **(b, d, f, h)**. The mutations in **e-h** were carried out on the background of the *Mt*PIPS-FL WT construct with *Af*2299 fusion and interface mutations.

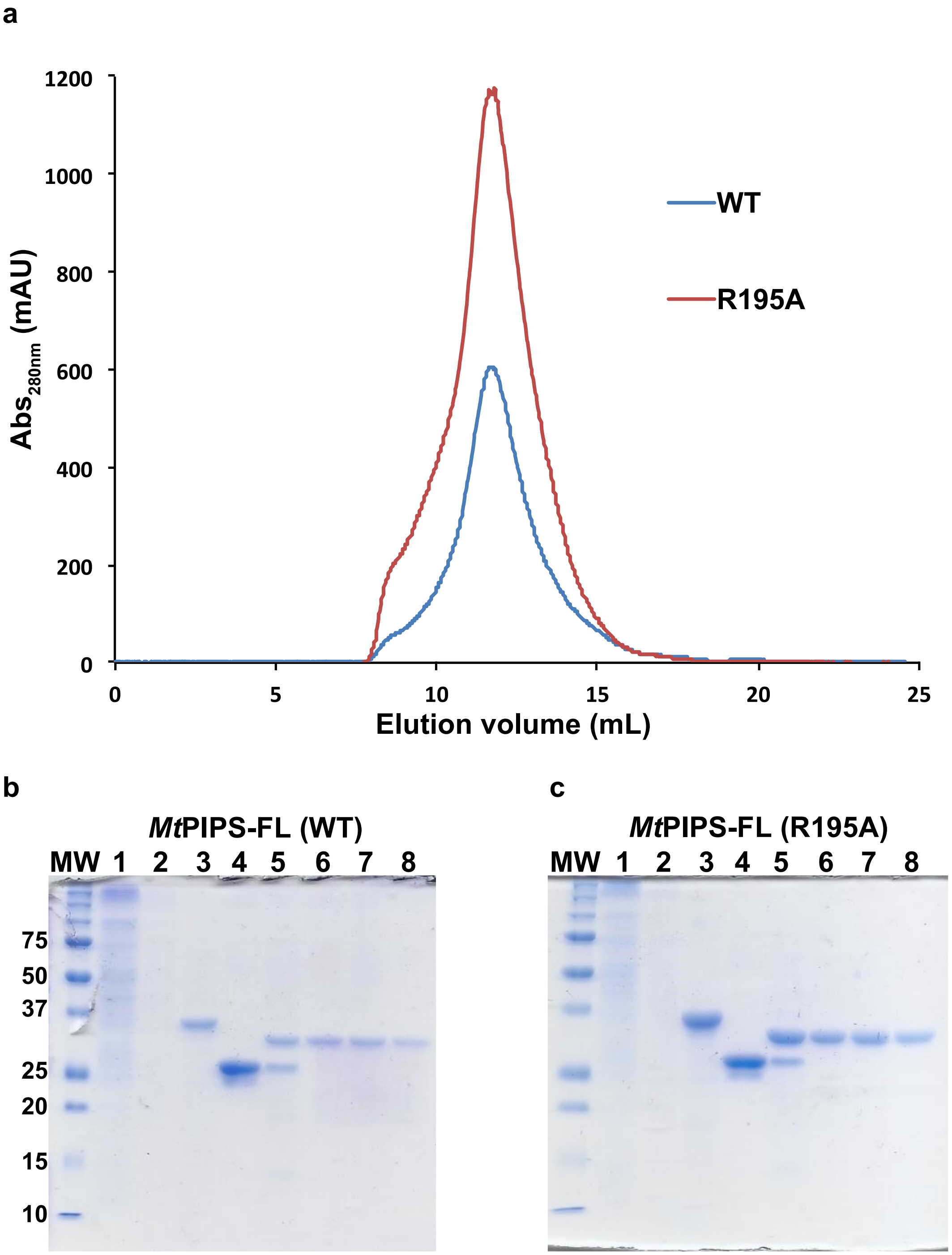
a
Abs280nm (mAU)
1200
1000
800
600
400
200
0
0
5
10
15
20
25
Elution volume (mL)
WT
R195A
b
MtPIPS-FL (WT)
MW 1 2 3 4 5 6 7 8
75
50
37
25
20
15
10
c
MtPIPS-FL (R195A)
MW 1 2 3 4 5 6 7 8

Supplementary Figure 7 | The R195A point mutation does not compromise folding of *Mt*PIPS-FL. **(a)** The wild-type and mutant proteins purified from isolated membrane fractions elute at the same volume when loaded on a size-exclusion chromatography column (SEC; Sephadex S200, GE). **(b)** and **(c)** present SDS-PAGE analysis of fractions collected during the purification of the WT and mutant proteins, respectively. Lane MW contains the molecular weight markers, with sizes indicated in kDa; lane 1 contains the flow-through from the metal-affinity chromatography (IMAC) column; lane 2 contains the wash; lane 3 contains the uncut elution from the IMAC column; lane 4 contains a sample of the purified TEV protease used for His-tag removal; lane 5 contains the crude cleaved eluate; lanes 6-8 contain samples of the three central fractions of the SEC, after reverse purification to remove uncleaved protein.

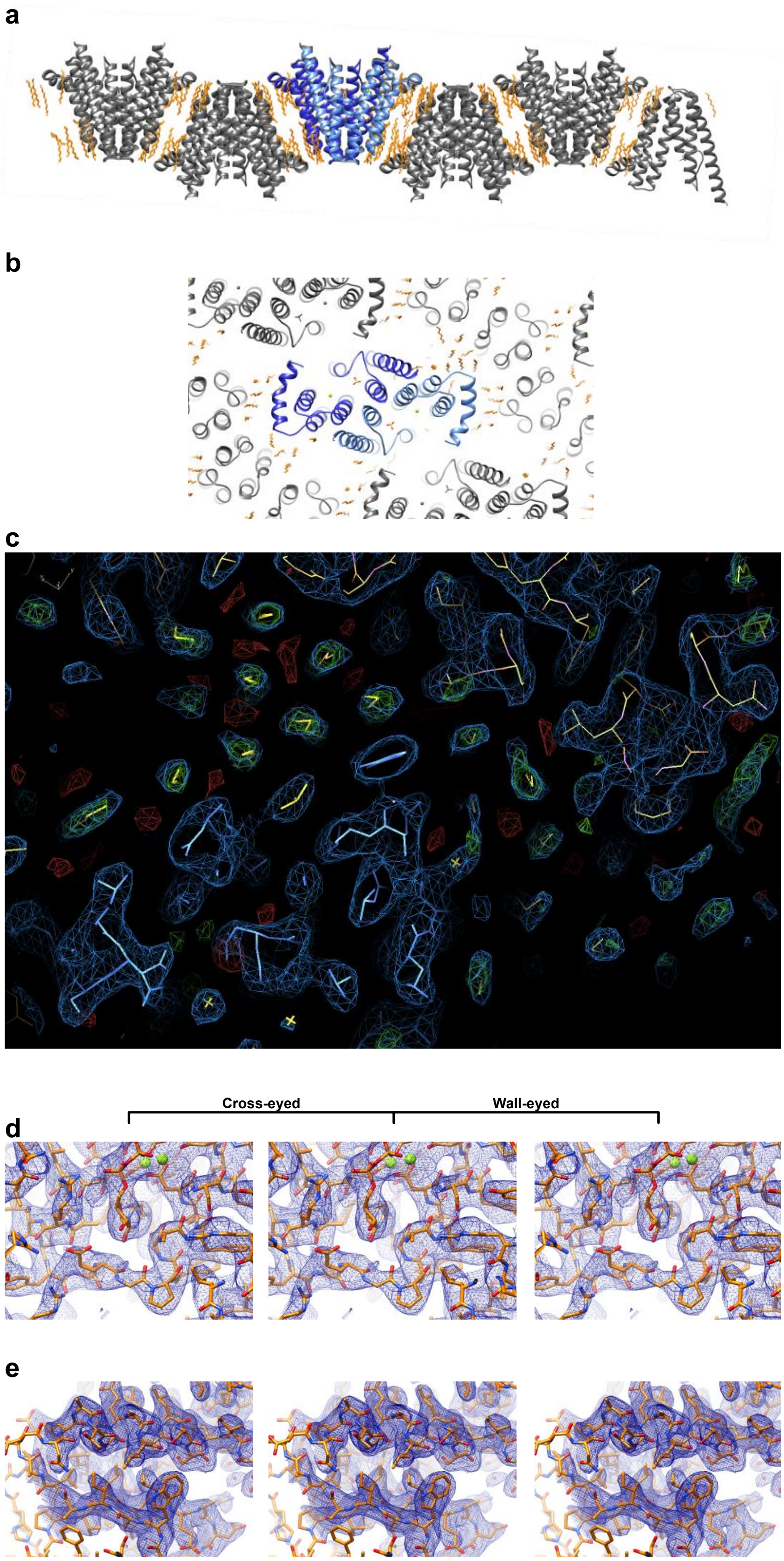
a
b
c
Cross-eyed
Wall-eyed
d
e

Supplementary Figure 8 | In the crystal lattice, the transmembrane domain of *Rs*PIPS is embedded in a hexagonal array of lipid acyl chains. The transmembrane domains of several adjacent *Rs*PIPS protomers are shown viewed parallel to the membrane in **(a)**, and from the cytosol in **(b)**. The two protomers comprising the central homodimer are represented in contrasting shades of blue, and partial lipids are depicted in orange stick representation. All other protomers are colored gray. **(c)** A slab through $2mF_o$-DF_c (blue mesh, contoured at 1.0XR.M.S.) and mF_o-DF_c (green/red mesh, contoured at 3.0XR.M.S.) maps calculated prior to the placement of lipid shows strong density for many lipid acyl chains. Representative sections of the final $2mF_o$-DF_c maps are shown contoured at 1.0XR.M.S for the apo and CDP-DAG bound structures in panels **(d)** and **(e)** respectively.

Initial constructs		
Mycobacterium smegmatis (Q9F7Y9)	Forward	tccctcatcggttctggatcaTCCAATGTCTACCTGATGACC
	Reverse	atccgttatccacttccaatgTTACGGTTCGCTCGTTTCCGG
Mycobacterium abscessus (G6X547)	Forward	tccctcatcggttctggatcaAGTGGTCTGCTGTCCCGTGAA
	Reverse	atccgttatccacttccaatgTTACGGTTGATTCGTTTCGGC
Mycobacterium fortuitum subsp. fortuitum (K0UMF3)	Forward	tccctcatcggttctggatcaTCTAACCTGTTCCTGATGTCG
	Reverse	atccgttatccacttccaatgTTAAGACGCACCCGGATCCGT
Mycobacterium avium subsp. paratuberculosis (R4N892)	Forward	tccctcatcggttctggatcaAGTAAAGTGCCGTTCCTGAGC
	Reverse	atccgttatccacttccaatgTTACGGAGCATCGGAACCACG
Mycobacterium marinum (D5MTP6)	Forward	tccctcatcggttctggatcaTCGAAAGCCCCGTTCCTGAGT
	Reverse	atccgttatccacttccaatgTTAGCGTTGCGTTTTACCCGG
Mycobacterium tuberculosis (Q7D6W6)	Forward	tccctcatcggttctggatcaTCGAAACTGCCGTTCCTGTCT
	Reverse	atccgttatccacttccaatgTTAGCGATCACCTTTGCCCGG
Gordonia polyisoprenivorans (H6MZX4)	Forward	tccctcatcggttctggatcaCTGTCCATTCGTGGTCGTGCC
	Reverse	atccgttatccacttccaatgTTAGCGTGCATCTGCACCCGG
Rhodococcus sp. (strain RHA1) (Q0S1E0)	Forward	tccctcatcggttctggatcaCTGTCGTTCTTCGGTCGTGCC
	Reverse	atccgttatccacttccaatgTTATTGCACGCTTGCCTGGCC
Nocardia farcinica (Q5YTD3)	Forward	tccctcatcggttctggatcaCTGTCGTTCTTCGGTCGTGAA
	Reverse	atccgttatccacttccaatgTTAGCGCGCTTCACCCGCAGC
Streptomyces sp. AA4 (D9UX52)	Forward	tccctcatcggttctggatcaCTGAATATCTTTGCTCGTGCC
	Reverse	atccgttatccacttccaatgTTACGGTTGGGCACCGCCCGC
Microlunatus phosphovorus (F5XFI2)	Forward	tccctcatcggttctggatcaCTGGAACGCTTTCGTGCTGGT
	Reverse	atccgttatccacttccaatgTTACGGAGCTTTTGCTTGACG
Brevibacterium casei (K9B2F1)	Forward	tccctcatcggttctggatcaCTGAATACCATTGCCCGTGCC
	Reverse	atccgttatccacttccaatgTTAGCTGGTTTCGTGGTTTTC
Renibacterium salmoninarum (A9WSF5)	Forward	tccctcatcggttctggatcaCTGAATAAATACGCCCGTGGT
	Reverse	atccgttatccacttccaatgTTAGGAAGCGGTCCAAGCTTT
Janibacter hoylei (K1ENZ2)	Forward	tccctcatcggttctggatcaCTGAATCGTTATGCTCGTGCT
	Reverse	atccgttatccacttccaatgTTAGGACGTCGCCGGGGTACT
*Af*2299 vector	Forward	CATTGGAAGTGGATAACGGAT
	Reverse	tgatccagaaccGATGAGGGAGCTCAGAAGGAG

*Rs*PIPS and *Mt*PIPS deletion constructs		
*Rs*PIPS-GS	Forward	CTGAGCTCCCTCATCGGTTCACTGAATAAATACGCC
	Reverse	GGCGTATTTATTCAGTGAACCGATGAGGGAGCTCAG
*Rs*PIPS-Δ6N	Forward	ATCGGTTCTCGTGGTCTGTTCGCTGCTATC
	Reverse	CAGACCACGAGAACCGATGAGGGAGCTCAG
*Mt*PIPS-Δ6N (with GS linker)	Forward	ATCGGTTCTCGCGCTGCTTTTCGTCGTATCACC
	Reverse	AGCGCGAGAACCGATGAGGGAGCTCAGAAGGAG

*Mt*PIPS with no fusion or interface mutations		
*Mt*PIPS-FL WT (for amplification from *Mt* genomic DNA)	Forward	TTCCAATCCAATGCCATGAGCAAGCTGCCCTTC
	Reverse	TATCCACTTCCAATGTCACCGGTCGCCCTTTCC
pMCSG7 vector	Forward	CATTGGAAGTGGATAACGGATCCG
	Reverse	GGCATTGGATTGGAAGTACAGGTT

*Mt*PIPS mutants for functional studies		
*Mt*PIPS-D31A	Forward	ATGTTGGTCTGACGCCGGCCGTGGTTACCATTCTG
	Reverse	CAGAATGGTAACCACGGCCGGCGTCAGACCAACAT
*Mt*PIPS-M69A	Forward	CGTGCTGTTTGATGCGCTGGACGGTGCAATGGCA
	Reverse	TGCCATTGCACCGTCCAGCGCATCAAACAGCACG
*Mt*PIPS-M69F	Forward	TTCGTGCTGTTTGATTTCCTGGACGGTGCAATG
	Reverse	CATTGCACCGTCCAGGAAATCAAACAGCACGAA

*Mt*PIPS-M69W	Forward	TTCGTGCTGTTTGATTGGCTGGACGGTGCAATG
	Reverse	CATTGCACCGTCCAGCCAATCAAACAGCACGAA
*Mt*PIPS-L70W	Forward	GTGCTGTTTGATATGTGGGACGGTGCAATGGC
	Reverse	GCCATTGCACCGTCCCACATATCAAACAGCAC
*Mt*PIPS-D93N	Forward	CTGGATGCGACGTGCAACCGCATTTCTGATGGC
	Reverse	GCCATCAGAAATGCGGTTGCACGTCGCATCCAG
*Mt*PIPS-S132A	Forward	GACGAGCCAGGTTATTGCTTATATCAAAGCAC
	Reverse	GTGCTTTGATATAAGCAATAACCTGGCTCGTC
*Mt*PIPS-K135A	Forward	GGTTATTTCTTATATCGCAGCACGTGCTGAAGC
	Reverse	GCTTCAGCACGTGCTGCGATATAAGAAATAACC
*Mt*PIPS-P153A	Forward	GGTTTCATTGAACGTGCGGAACGCCTGATTATC
	Reverse	GATAATCAGGCGTTCCGCACGTTCAATGAAACC
*Mt*PIPS-P153V	Forward	GGTTTCATTGAACGTGTTGAACGCCTGATTATC
	Reverse	GATAATCAGGCGTTCAACACGTTCAATGAAACC
*Mt*PIPS-P153W	Forward	GGTTTCATTGAACGTTGGGAACGCCTGATTATC
	Reverse	GATAATCAGGCGTTCCCAACGTTCAATGAAACC
*Mt*PIPS-R155A	Forward	CATTGAACGTCCGGAAGCCCTGATTATCGTCCTG
	Reverse	CAGGACGATAATCAGGGCTTCCGGACGTTCAATG
*Mt*PIPS-R155Q	Forward	ATTGAACGTCCGGAACAGCTGATTATCGTCCTG
	Reverse	CAGGACGATAATCAGCTGTTCCGGACGTTCAAT
*Mt*PIPS-R195A	Forward	CATCACCTGCGTCCAAGCTCTGCACACCGTGTGG
	Reverse	CCACACGGTGTGCAGAGCTTGGACGCAGGTGATG
*Mt*PIPS-R195Q	Forward	ATCACCTGCGTCCAACAGCTGCACACCGTGTGG
	Reverse	CCACACGGTGTGCAGCTGTTGGACGCAGGTGAT

Supplementary Table 1 | Primers used for cloning and mutagenesis. Sequences are provided for primers used in initial cloning of *Af*2299-PIPS (full-length) fusions and in site-directed mutagenesis to generate the additional *Rs*PIPS and *Mt*PIPS constructs. Regarding the primers used to produce the initial constructs, upper case letters indicate gene-specific sequences and lower case letters indicate sequences incorporated into the PCR product to generate the overlaps necessary for Gibson assembly. All primers are written from 5’ to 3’.

Supplementary References

1. Pei, J., Kim, B.-H. & Grishin, N. V. PROMALS3D: a tool for multiple protein sequence and structure alignments. *Nucleic Acids Research* **36,** 2295–2300 (2008).
2. Krogh, A., Larsson, B., Heijne, von, G. & Sonnhammer, E. L. Predicting transmembrane

protein topology with a hidden Markov model: application to complete genomes. *J. Mol. Biol.* **305,** 567–580 (2001).

3. Pei, J., Kim, B. H. & Grishin, N. V. PROMALS3D: a tool for multiple protein sequence and structure alignments. *Nucleic Acids Research* **36,** 2295–2300 (2008).
